# DeCTCF: Decoding CTCF binding sequences by leveraging predicted epigenomic features

**DOI:** 10.1371/journal.pcbi.1014848

**Published:** 2026-10-05

**Authors:** Lu Chai, Jie Gao, Tinghe Guo, Teer Ba, Zihan Li, Junjie Liu, Yong Wang, Lirong Zhang

**Affiliations:** 1 School of Physical Science and Technology, Inner Mongolia University, Hohhot, China; 2 CEMS, NCMIS, HCMS, MDIS, Academy of Mathematics and Systems Science, Chinese Academy of Sciences, Beijing, P. R. China; 3 Inner Mongolia Key Laboratory of Biophysics and Bioinformatics, Inner Mongolia University, Hohhot, China; University of Queensland, AUSTRALIA

## Abstract

CTCF is a key architectural protein with diverse roles in genome organization and gene regulation, yet how it achieves these roles in different contexts remains unclear. Pretrained sequence-based models such as Sei provide predicted epigenomic features that can be used in downstream analyses of regulatory elements. Here, we developed DeCTCF, an integrative computational framework that uses pretrained Sei predictions to analyze 236,552 CTCF binding sequences by integrating CTCF ChIP-seq data from 118 human cell lines. By leveraging predicted epigenomic features from the Sei model, we grouped these CTCF binding sites into 20 clusters. These clusters can be annotated into distinct functional modules, including a major module associated with 3D chromatin architecture and three lineage-associated modules. The lineage-associated modules reveal associations between candidate co-factors and CTCF’s context-dependent functions. For example, several clusters enriched in the three stem cell lines included in our dataset also showed enrichment of ZIC-family and were associated with gene sets related to pluripotency and neurodevelopment. We further observed associations between cluster-level CTCF ChIP-seq signal profiles and chromatin-loop annotations: single-peak profiles were reproducibly associated with higher loop interaction scores, whereas double- and triple-peak profiles showed distinct loop-pairing preferences. Overall, our study offers a systematic map of CTCF’s modular organization by leveraging predicted epigenomic features and reveals context-associated regulatory patterns that underlie its regulatory diversity.

## Author summary

In human cells, transcription factors regulate gene expression through diverse mechanisms. Given that CTCF is a ubiquitous factor with diverse roles in chromatin organization and gene regulation, it remains unclear how it achieves functional diversity despite binding to a conserved DNA motif. To address this, we developed a computational framework, DeCTCF. It leverages epigenomic features predicted by the pretrained Sei model to analyse over 230,000 CTCF binding sequences across 118 human cell lines and systematically classifies these sites into 20 clusters. These clusters were further grouped into four functional modules, including three cell-type-associated regulatory modules and one chromatin organization-associated module. We find that the context-associated regulatory functions of CTCF are associated with lineage-specific transcription factors and distinct epigenomic environments. In contrast, its role in chromatin organization is associated with CTCF ChIP-seq signal profiles and interactions with co-factors, which together may contribute to the patterns of chromatin looping. Together, our results provide a generalizable framework for decoding the functional diversity of regulatory elements from sequence and epigenomic features.

## Introduction

Transcription factors (TFs) are DNA-binding proteins that play key roles in gene regulation [1]. The human genome is estimated to encode approximately 1,639 TFs [2], and CCCTC-binding factor (CTCF) is one of the most extensively studied proteins, ubiquitously present in nearly all vertebrate tissues [3,4]. As a multifunctional TF, CTCF has long been recognized for its significant roles in transcriptional regulation and chromatin organization [5]. Early studies have highlighted the multifaceted regulatory roles of CTCF, such as acting as an insulator that mediates enhancer blocking [6–8], promoting the inclusion of weak upstream exons through RNA polymerase II pausing during splicing [9,10], activating transcription at specific promoters [11,12], and orchestrating three-dimensional genome organization via chromatin loop formation [13,14]. The advent of high-throughput sequencing technologies, particularly ChIP-seq and Hi-C, has greatly enhanced our understanding of CTCF’s genomic functions [15,16]. Genomic studies have demonstrated that CTCF binding is highly enriched at the boundaries of most topologically associating domains (TADs), which are crucial for transcriptional insulation [17]. More recently, single-cell multi-omics studies have revealed that CTCF executes distinct functions in different cellular contexts, with cell type-specific differences in its binding patterns and accessibility [18–22].

Recent studies have revealed that CTCF binding is dynamic and context-dependent, with distinct roles emerging across various cell types and biological processes. For example, in CD8+ T cells, weak-affinity CTCF binding promotes terminal differentiation by regulating key transcriptional programs [23]. In oncogenic T-cell acute lymphoblastic leukemia, NOTCH1 induces specific CTCF binding, which cooperatively activates target gene expression [24]. CTCF binding also plays a critical role in cell cycle regulation, embryonic development, and the formation of various adult cell types [25–28]. Furthermore, in prostate cancer, CTCF co-binding with MYC rewires chromatin architecture, highlighting its involvement in oncogenesis [29]. Moreover, alterations in CTCF-mediated 3D chromatin organization have been implicated in Alzheimer’s disease, with potential links to diminished target gene expression and changes in histone modifications [30]. Taken together, these studies underscore the functional diversity of CTCF binding. However, the precise mechanisms through which it mediates these context-dependent roles remain poorly understood.

Recent advances in deep learning have transformed our capacity to decode the functional grammar of regulatory elements [31]. Traditional approaches relying on motif scanning or chromatin accessibility measurements often fail to capture the combinatorial logic governing TF binding specificity [32,33]. Foundation models like Basenji2 [34] and transformer-based models like Enformer [35] learn base-pair-level sequence determinants by leveraging massive genomic datasets, whereas graph neural networks excel at modeling three-dimensional chromatin contacts as relational graphs [36]. Importantly, models such as Sei [37] integrate raw DNA sequences with large-scale chromatin state maps to learn unified embeddings of regulatory potential, thereby enabling quantitative inference of TF context-associated regulatory activity directly from DNA sequence. Building on Sei’s comprehensive vocabulary, we apply a graph-based clustering strategy to predicted epigenomic features to systematically dissect the functional diversity of CTCF binding sequences (CBSs).

Here, we present DeCTCF, an integrative computational framework designed to systematically decode the functional diversity of CBSs. Using epigenomic features predicted by the Sei model, over 230,000 CBSs from 118 human cell lines were embedded into high-dimensional feature vectors, and classified into 20 distinct clusters, which were further organized into four important function-associated modules. First, we identified a chromatin architecture-associated module (CAM), comprising Clusters 2, 3, and 16, which revealed three potential patterns involving the chromatin-loop organization. Next, we characterized two lineage-associated modules, a stem cell-enriched module (SCM) and an immune cell-enriched module (ICM). The SCM reveals two major regulatory modes. One consists of Cluster 1, 12 and 17 which are characterized by enrichment of ZIC-family motifs and association with neurodevelopment-related gene sets. The other contains Clusters 6, 8, and 13, which are associated with pluripotency and lineage priming. The ICM delineates three groups of CBSs in immune cells which consist of Cluster 5, 15 and 18. Finally, we defined the TF cooperation module (TFCoM) comprising Cluster 4, 7, 9, 10, 11, and 14. This module shows that the candidate co-factors of each cluster are linked to specific local histone/chromatin-accessibility signatures and characteristic CTCF/cohesin peak shapes. Furthermore, we discovered a link between CTCF ChIP-seq signal architecture and function, showing that single-peak profiles are associated with stable loop anchors, whereas multi-peak profiles occur in distinct regulatory contexts. Ultimately, DeCTCF provides a comprehensive framework for characterizing CTCF binding, offering mechanistic insights into the sequence determinants that may contribute to its regulatory diversity.

## Results

### Overview of DeCTCF

Our study aims to systematically decode the functional diversity of CBSs, providing a comprehensive atlas of how its various roles are associated with different cellular contexts (Fig 1A). To achieve this, we developed DeCTCF, an integrative computational framework. This framework represents each CBS using high-dimensional epigenomic feature vectors predicted by the Sei model, reflecting its sequence-derived regulatory potential. By integrating these high-dimensional features with a graph-based clustering algorithm, the CBSs were partitioned into functionally coherent clusters. Each resulting cluster was then systematically characterized using four annotation dimensions to determine its unique biological properties.

**Fig 1:**
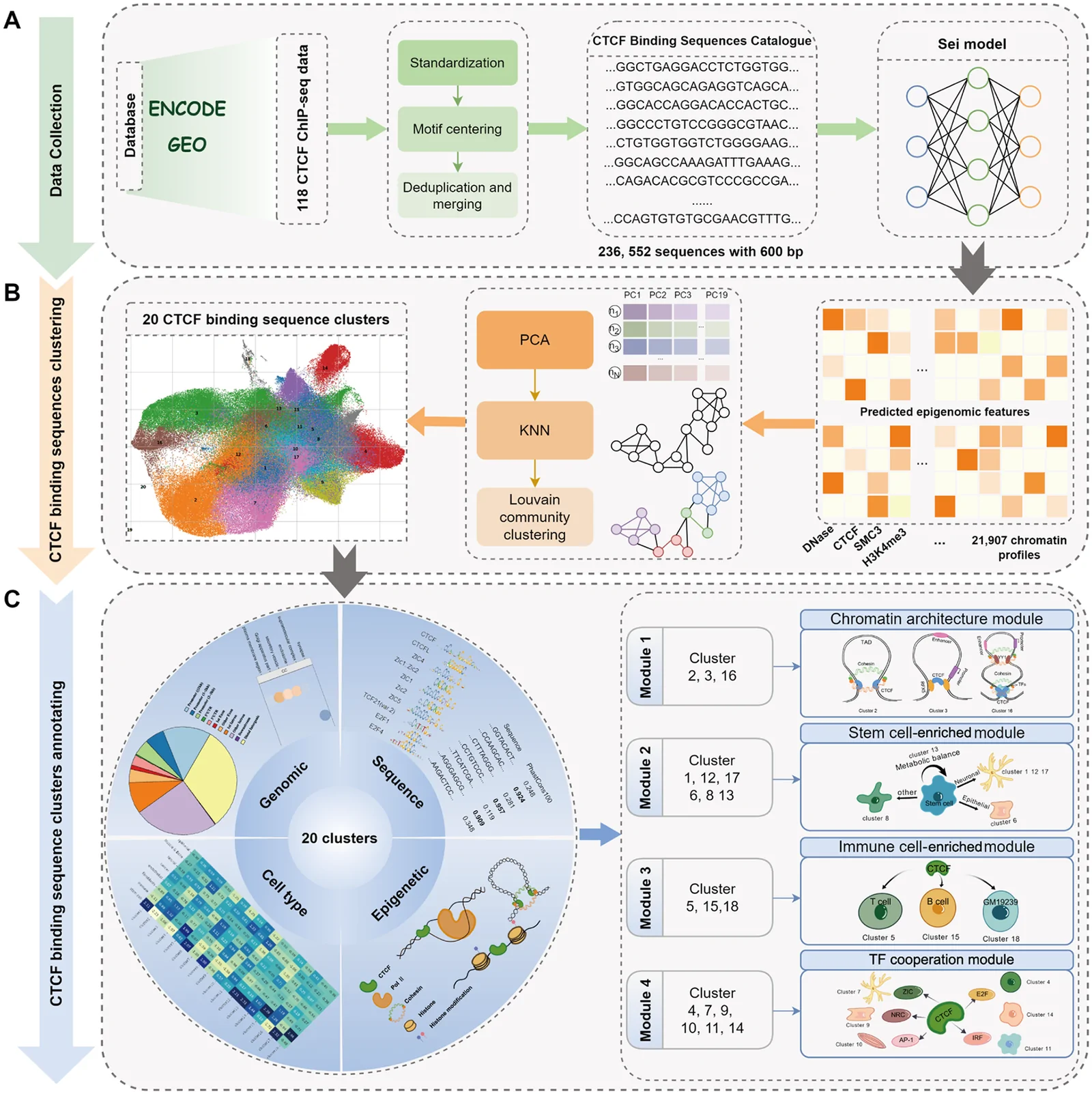
Overview of the DeCTCF framework. (A) Data collection and processing pipeline. A comprehensive catalogue of 236,552 unique CBSs was constructed by aggregating and processing 118 human ChIP-seq datasets from ENCODE and GEO. (B) Graph-based clustering of CTCF binding sequences. CTCF binding sequences were partitioned into 20 clusters via a graph-based clustering pipeline (PCA, KNN, and Louvain community) leveraging epigenomic features predicted by the pretrained Sei model. (C) Annotation and module definition. The 20 clusters were characterized across four annotation dimensions and organized into four major modules.

Applying DeCTCF to a comprehensive catalogue of 236,552 CBSs derived from 118 human cell lines (S1 Table), we classified this catalogue of CBSs into 20 distinct clusters (Fig 1B). Given that CTCF’s binding events are central to genome architecture and cell identity, our systematic clustering and annotation provide a resource for investigating associations between CTCF binding and biological processes such as 3D genome organization, stem cell differentiation, and immune cell function (Fig 1C). The numbers and proportions of CBSs assigned to the individual clusters and higher-order modules are summarized in S2 Table. This foundational map provides a basis for describing distinct regulatory modes of CBSs and investigating the organizing principles underlying their modular organization.

### CTCF shaping chromatin structure with distinct binding modes

Applying unsupervised Louvain community clustering, we divided CBSs into 20 distinct clusters. To explore the role of these CBSs in 3D genome organization, we first assessed cluster enrichment at TAD boundaries, which revealed that Clusters 2, 3, and 16 showed the strongest enrichment (z-scores: 1.42, 1.06, and 1.56, respectively; Fig 2C). Accordingly, we designated these three clusters as the CAM and examined their distinct mechanisms in shaping chromatin architecture.

**Fig 2:**
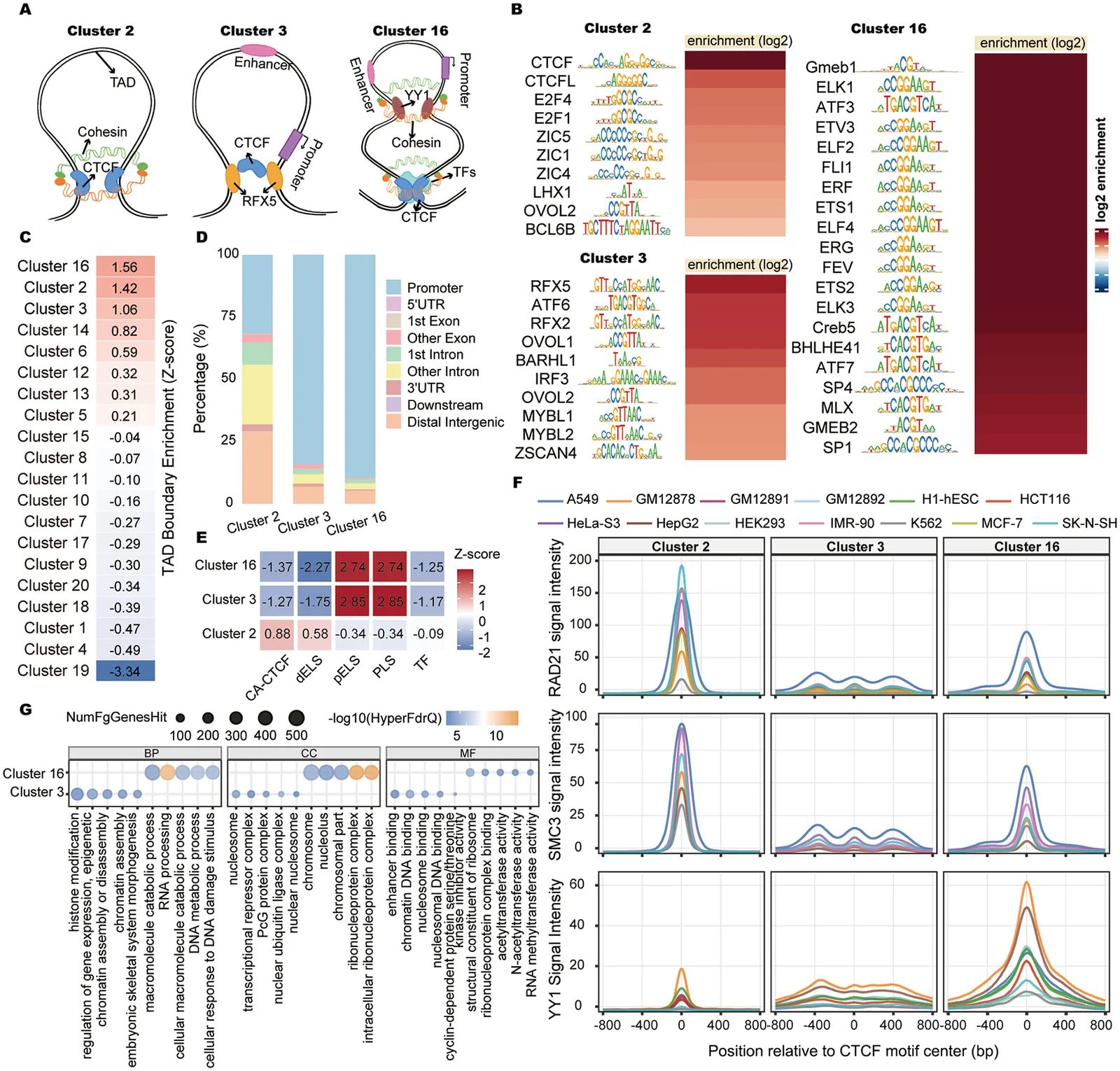
Functional characterization of CTCF binding sequence clusters associated with chromatin architecture. (A) Three candidate modes associated with CTCF-mediated chromatin organization: a canonical Cohesin-associated mode (Cluster 2), an RFX5 motif-associated promoter-enhancer mode (Cluster 3), and a co-factor motif-associated mode involving Gmeb1 and YY1 (Cluster 16). (B) De novo motif enrichment for key TFs. Heatmaps display log2 enrichment scores of key TFs. (C) Heatmap of TAD boundary enrichment Z-scores for all 20 CTCF clusters. Cluster 2, 3, and 16 show the highest positive enrichment, indicating a strong association with TAD boundaries. (D) Stacked bar plot of genomic feature annotations for Cluster 2, 3, and 16. Different colors represent distinct functional genomic categories, and the y-axis indicates the percentage of each category within the clusters. (E) Heatmap showing the enrichment Z-score of clusters within different SCREEN candidate Cis-Regulatory Elements. (F) Profiles of RAD21, SMC3, and YY1 binding signals. The x-axis displays the distance from the motif center of CBSs, while the y-axis represents the ChIP-seq signal intensity for each transcription factor. Different colored curves indicate distinct cell lines. (G) Bubble plot showing Gene Ontology (GO) enrichment analysis for Cluster 3 and 16. The size of each dot represents the number of foreground genes associated with each cluster, while the color corresponds to the -log10(FDR q-value) with a threshold of q < 0.05.

Cluster 2 contains 23,029 CBSs characterized by exceptionally high sequence conservation. Consistent with this score, the sites in Cluster 2 are occupied across nearly all 118 cell lines (S1 Fig). This cluster had the lowest cell-line specificity score of 0.13 among all clusters (S2A Fig, methods). Furthermore, phastCons analysis demonstrated significant enrichment of conserved elements (phastCons > 0.8; p << 1 × 10^−5^), indicating strong evolutionary constraint (S2B Fig). Through de novo motif analysis, we verified a strong enrichment of the CTCF motif at these loci (Fig 2B). RAD21 exhibited a central peak in 10 cell lines; SMC3 mirrored this pattern, whereas YY1 signals were negligible (Fig 2F). In the ranking of 21,907 epigenomic features derived from the Sei model, 48 of the top 50 were features of DNase peaks (S3 Table). Together, these results characterize Cluster 2 as a group of evolutionarily conserved CBSs. These CBSs in Cluster 2 are anchored by strong CTCF and show strong cohesin occupancy, supporting their roles as stable architectural anchors. Here, CTCF binds directly to conserved loci via its canonical motif which is co-occupied by cohesin (RAD21/SMC3), and helps detect anchors of stable chromatin loops enriched at most TAD boundaries. The CTCF/cohesin ChIP-seq signal profiles of these CBSs are consistent with the “classical” loop extrusion model, in which CTCF-cohesin complexes serve as stable structural anchors for genome folding (Fig 2A).

Cluster 3 contains 21,935 CBSs characterized by strong RFX5 motif enrichment and limited relative enrichment of the canonical CTCF motif (Fig 2B). In contrast to Cluster 2 and 16, the ChIP-seq signals of RAD21 and SMC3 at the CBSs in Cluster 3 were substantially lower, suggesting distinct organizational rules underlying chromatin architecture (Fig 2A). ChIPseeker genome annotation indicated that the majority of the CBSs in Cluster 3 (80.77%) are promoter-proximal (Fig 2D). Consistently, the CBSs of Cluster 3 were significantly enriched for proximal enhancer-like (pELS; z = 2.85) and promoter-like (PLS; z = 2.74) elements based on cCREs (hg38) analysis from SCREEN (Fig 2E). GO enrichment analysis suggested that Cluster 3 is involved in regulatory programs linked to higher-order chromatin architecture, with terms including “histone modification,” “chromatin assembly,” and “epigenetic regulation of gene expression” (Fig 2G). This observation was supported by Sei model feature ranking, which showed a strong dominance of promoter-associated marks like H3K4me3 (S3 Table). To confirm these findings with respect to motif enrichment, we further analyzed publicly available RFX5 ChIP-seq datasets from HeLa-S3, HepG2, IMR-90, and SK-N-SH cell lines. The results indicate a consistent enrichment regarding RFX5 ChIP-seq signals around Cluster 3 CBSs in the four cell lines (S4 Fig), providing occupancy-based evidence for an association between RFX5 and Cluster 3 CBSs. These observations suggest that RFX5 could be a candidate co-factor associated with Cluster 3. Therefore, Cluster 3 can be inferred to be a group of promoter-proximal CBSs enriched for RFX5 motifs. Although the enrichment of RFX5 motifs suggests a potential association between RFX5 and CTCF occupancy at these sites, direct recruitment and its effects on promoter-enhancer contacts require further validation. In this mode, CTCF binding may be associated with transcriptionally poised higher-order structures despite comparatively weak enrichment of the canonical CTCF motif and low cohesin occupancy, potentially linking developmental signals to 3D genome folding (Fig 2A).

Cluster 16 comprises 6,529 CBSs exhibiting significant enrichment of Gmeb1 and other high‐GC TF motifs but comparatively weak enrichment of the canonical CTCF motif (Fig 2B). The ChIP–seq signals of RAD21, SMC3 and YY1 exhibit a central peak (±200 bp) around these CBSs (Fig 2F). Similar to Cluster 3, genomic annotation indicated that the majority of Cluster 16 loci (87.3%) are promoter-proximal (Fig 2D). This observation was further supported by SCREEN cCRE data, which showed significant enrichment for promoter-like (PLS) and proximal enhancer-like (pELS) elements (Fig 2E). Epigenomic feature prioritization by the Sei model highlighted chromatin accessibility and promoter activity as defining signatures: DNase I and H3K4me3 accounted for 30 and 14 of the top 50 ranked features, with additional contributions from H3K4me2 and H3K27ac. These marks were consistently derived from embryonic, neuronal, and proliferative cell contexts, underscoring the broad functional relevance of this cluster (S3 Table). GO enrichment analysis pointed to an association between Cluster 16 and nuclear pathways such as RNA/DNA metabolism and enzymatic functions (Fig 2G). Together, these findings characterize Cluster 16 as a promoter-proximal subset enriched for Gmeb1 motifs. Mechanistically, the analysis of motif enrichment and occupancy patterns suggests potential interactions of Gmeb1 and YY1 with CTCF at these CBSs. Architecturally, its co-occupancy with cohesin may be associated with dynamic chromatin loops. Functionally, these CBSs provide active centers for the metabolic and enzymatic processes, as supported by the GO analysis. This potential TF-associated mode may link CTCF binding and genome architecture with essential nuclear functions, despite the limited relative enrichment of the canonical CTCF motif (Fig 2A).

### Stem cell-enriched CTCF regulatory patterns

By analyzing cell type associations across all clusters, we identified a stem cell-enriched module (SCM), which reveals two major regulatory modes (Figs 3-4). The first mode is characterized by the co-enrichment of ZIC-family motifs and is broadly associated with neurodevelopment. This module consists of Cluster 1, 12, and 17, which are preferentially enriched in the three stem cell lines included in our dataset (z-scores > 2.1; Fig 3A) and together account for 15.36% of all profiled CBSs. Protein–protein interaction mapping further connected ZIC1 and ZIC5 to FOXD3 and ZPR1, forming a compact network of neural regulators associated with CTCF (Fig 3B). The three clusters also displayed distinct aggregate CTCF ChIP-seq signal profiles. Cluster 1 and 12 exhibit a canonical single-peak CTCF ChIP-seq signal profile, whereas Cluster 17 displays a distinct triple-peak profile, suggesting a more complex regulatory context (Fig 3C). Motif enrichment analysis revealed strong co-enrichment of ZIC-family motifs across all three clusters (Fig 3D). Cluster 17 possesses a more diverse ZIC-family motif signature, with specific enrichment for ZIC3, a known core TF in embryonic stem cells, further supporting its distinct stem cell-related regulation role [38]. Together, these findings suggest potential cooperation between CTCF and ZIC-family TFs in the three stem cell lines, with links to neurodevelopment-related regulatory programs.

**Fig 3:**
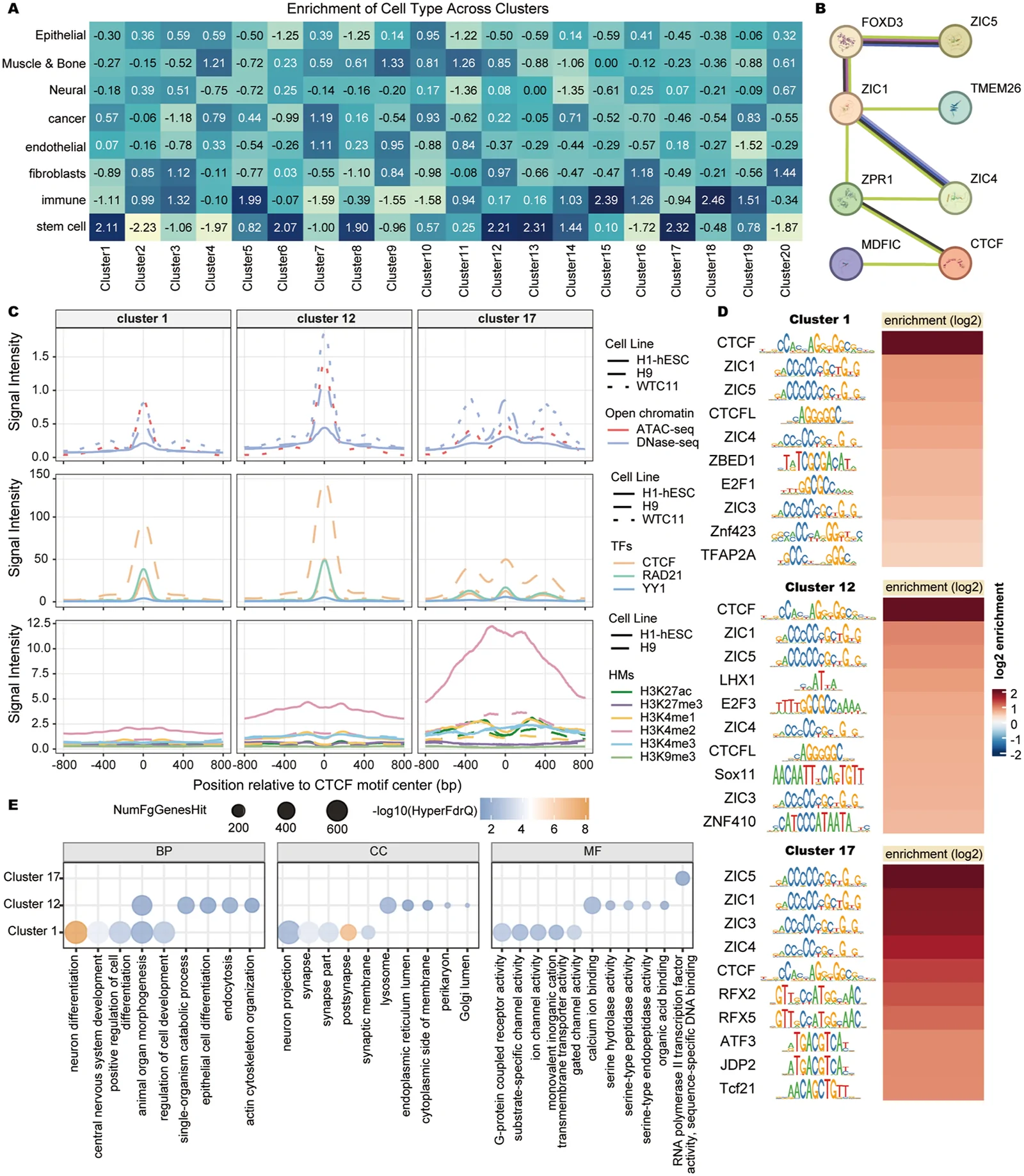
Neurodevelopment-related CTCF regulatory patterns in the stem cell-enriched module. (A) Heatmap of normalized enrichment Z-scores of cell type across all clusters. (B) Protein-protein interaction (PPI) network between CTCF and ZIC family transcription factors. The interacting factors shown (e.g., FOXD3, TMEM26) are predominantly associated with neural regulation. (C) Epigenomic profiles centered on CBSs (±800 bp). The x-axis displays the distance from the motif center of CBSs, while the y-axis represents the signal intensity of chromatin accessibility or epigenetic modifications. The top shows chromatin accessibility signals across the three stem cell lines; the middle displays ChIP-seq signals of CTCF, RAD21, and YY1; and the bottom presents ChIP-seq signals of six HMs. Distinct line styles correspond to different cell lines, and different colors indicate distinct epigenetic signals. (D) De novo motif enrichment for key transcription factors in each cluster. Heatmaps show log2 enrichment scores. ZIC-family motifs (e.g., ZIC1, ZIC2, ZIC3, ZIC5) are significantly enriched across all three clusters. (E) Bubble plot showing Gene Ontology (GO) enrichment analysis for Cluster 1, 12 and 17. The size of each dot represents the number of foreground genes associated with each cluster, while the color corresponds to the -log10(FDR q-value) with a threshold of q < 0.05.

**Fig 4:**
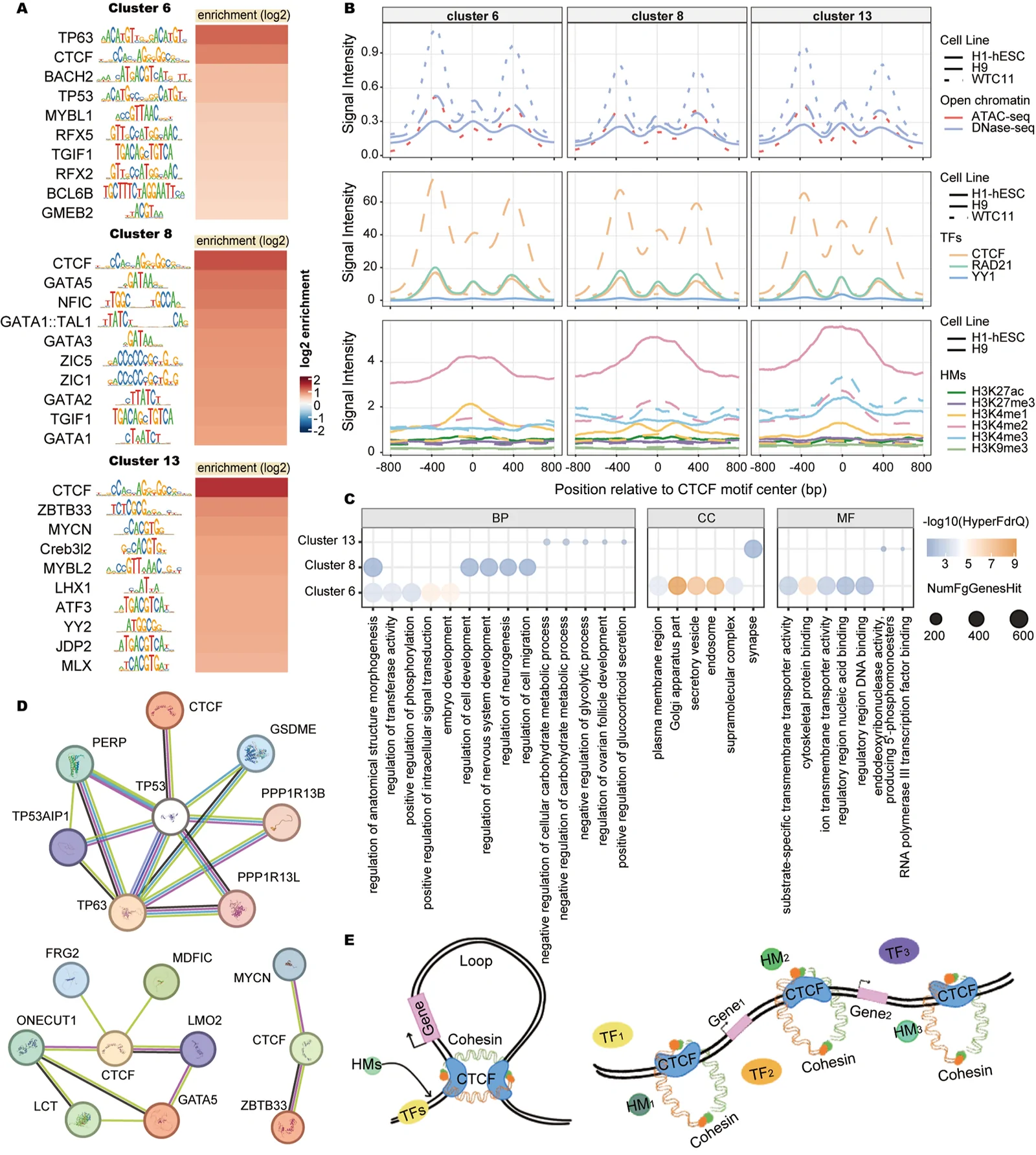
Divergent CTCF-associated regulatory programs in stem cell lines. (A) De novo motif enrichment for key transcription factors in each cluster. Heatmaps show log2 enrichment scores. (B) Epigenomic profiles centered on CBSs. The x-axis displays the distance from the motif center of CBSs, while the y-axis represents the signal intensity of chromatin accessibility or epigenetic modifications. The top shows chromatin accessibility signals across the three stem cell lines; the middle displays ChIP-seq signals of CTCF, RAD21, and YY1; and the bottom presents ChIP-seq signals of six HMs. Distinct line styles correspond to different cell lines, and different colors indicate distinct epigenetic signals. (C) Bubble plot showing Gene Ontology (GO) enrichment analysis for Clusters 6, 8 and 13. The size of each dot represents the number of foreground genes associated with each cluster, while the color corresponds to the -log10(FDR q-value) with a threshold of q < 0.05. (D) Protein-protein interaction (PPI) networks for the co-factors associated with each cluster: TP63 (Top, Cluster 6), GATA5 (Left bottom, Cluster 8), and MYCN/ZBTB33 (Right bottom, Cluster 13), showing reported interactions with CTCF and other partners. (E) Schematic illustrating two conceptual models for chromatin looping. Left: A conventional loop formed by a single cohesin complex. Right: A dimeric “handcuff” model where two cohesin rings are linked, consistent with the observed aggregate triple-peak profile of CTCF ChIP-seq signals.

Despite this shared foundation, the three clusters exhibit distinct functional associations, supported by their Gene Ontology (GO) profiles (Fig 3E). Cluster 1, the largest cluster (n = 23,309), is strongly associated with mature neuronal functions. Its biological processes include “neuron differentiation,” and cellular components map to the “synapse,” indicating a potential role in terminally differentiated neurons. In contrast, Cluster 12 (n = 10,240) is enriched for terms related to broader developmental processes and cellular organization. These terms include “animal organ morphogenesis” and “epithelial cell differentiation,” as well as components of the cell’s internal machinery, such as the “lysosome” and “endoplasmic reticulum.” Most notably, Cluster 17, the smallest cluster with the strongest stem cell association (n = 2,793), is uniquely linked to the fundamental molecular function of “RNA polymerase II TF activity,” connecting it with transcriptional regulatory processes related to the pluripotent state. Moreover, their functional differences are mediated by distinct chromatin states.

Taken together, the three clusters suggest a regulatory continuum spanning from the maintenance of core stem cell identity, related to Cluster 17’s distinct CTCF binding profile and ZIC3 enrichment, to lineage specification related to Cluster 12, and finally to terminal neuronal differentiation related to Cluster 1.

The second SCM mode comprises Cluster 6, 8, and 13, which partially overlap in the UMAP embedding and show functional associations with pluripotency maintenance and lineage priming processes (S3B Fig). The three clusters are all significantly enriched in the three stem cell lines, with z-scores of 2.07, 1.90, and 2.31, respectively. However, in contrast to the first mode, their functions are associated with distinct lineage-related TF motifs and divergent epigenetic landscapes. Motif analysis revealed that Cluster 6 is marked by TP63 and BACH2 motifs, Cluster 8 by GATA5 and NFIC motifs, and Cluster 13 by the pluripotency factor MYCN motif and the transcriptional repressor ZBTB33 motif (Fig 4A, 4D). Our analysis indicates that the enrichment of these TF motifs occurs in distinct epigenetic environments. Cluster 6 and 8 are dominated by active marks (H3K4me1, H3K27ac), suggesting that they are located at active or poised enhancers. In stark contrast, Cluster 13 is characterized by high levels of repressive marks (H3K9me3, H3K27me3), suggesting a silencing role (S3 Table). These diverse combinations of enriched motifs of lineage-related TFs and epigenetic states are associated with divergent biological functions, as further supported by GO analysis (Fig 4C). Therefore, we defined Cluster 6 as a category related to morphogenesis and signal transduction, Cluster 8 to nervous system development, and Cluster 13 to metabolic and hormonal regulation.

Interestingly, despite major differences in their functional associations and epigenetic marks, the CBSs in the three clusters of the second SCM mode share similar CTCF/cohesin ChIP-seq signal profiles across three stem cell lines. The aggregate ChIP-seq signal distributions of three core architectural proteins (CTCF, RAD21, and SMC3) show a triple-peak profile: a peak at the CBS center, flanked by two stronger peaks at approximately ±400 bp (Fig 4B). Open chromatin accessibility follows a similar pattern, though with lower signal intensity. This triple-peak profile is consistent with the “handcuff model”, which proposes that cohesin functions as a two-ring complex rather than a single ring [39]. Under this model, the two stronger flanking peaks could hypothetically reflect the positions of individual cohesin rings on the DNA, whereas the central peak could correspond to a linkage region between them. This distinct aggregate profile, also seen in Cluster 17, contrasts with the simpler single-peak profile of CTCF ChIP-seq signal of conventional loop anchors (Fig 4E). We propose the handcuff architecture as one possible interpretation of this aggregate profile. However, its relevance to chromatin looping and stem cell-enriched regulation requires validation in independently processed datasets and direct experiments.

### Immune cell-enriched CTCF regulatory patterns

In addition, through unsupervised clustering, we identified another module of CBSs that is particularly prominent in the immune cell lines included in our dataset (Fig 3A), indicating a potential association with immune cell identity and function. This immune cell-enriched module (ICM) consists of three clusters: Cluster 5 (7.40% of CBSs, z-score = 1.99), Cluster 15 (3.61%, z-score = 2.39), and Cluster 18 (0.47%, z-score = 2.46). Although these three clusters are located near each other in the UMAP embedding, they exhibit pronounced variation in regulatory features and functional associations.

First, the three clusters are distinguished from each other through de novo TF motif enrichment (Fig 5A). Cluster 5 is characterized by a dominant enrichment of the canonical CTCF motif, suggesting a potential structural role. In contrast, Cluster 15 was strongly co-enriched for key lymphocyte-associated TF motifs, including IRF4, NFKB1, and RELA, alongside CTCF. Cluster 18 is defined by a different set of lineage-associated motifs, most notably ZSCAN4 and the immune regulator BHLHE40. In Cluster 15, the co-occurrence of these motifs is supported by known protein-protein interactions (Fig 5B). NFKB1/RELA have been reported to cooperate with IRF family members at specific genomic loci, often within chromatin domains demarcated by CTCF, to drive robust gene expression. Using epigenomic data, we inferred that the diverse TF landscapes of Cluster 5, 15, and 18 correspond to their particular local chromatin states (Fig 5C). In GM12878 cells, both Cluster 5 and 18 display a canonical bimodal chromatin accessibility and cohesin ChIP-seq signal profiles, with a signal trough at the highest-scoring FIMO motif center and stronger flanking signals. This distribution may reflect altered local CTCF/cohesin occupancy caused by other transcription factors or regulatory complexes near the motif center, although its molecular basis and relationship to chromatin-loop anchoring remain unclear. It is accompanied by strong signals for the promoter-proximal histone mark H3K4me3. Cluster 15, however, exhibits a distinct aggregate signal profile. In contrast to the central trough observed in Cluster 5 and 18, Cluster 15 displays a triple-peak profile, with CTCF/cohesin peaks at positions -400 bp, + 400 bp, and the center (0 bp). The central peak is co-localized with IRF4 and RELA ChIP-seq signals and is accompanied by stronger signals for the active enhancer mark H3K27ac, in addition to H3K4me3. Furthermore, Cluster 18 shows enrichment of BHLHE40 binding, particularly in the GM12878 cell line (Fig 5C).

**Fig 5:**
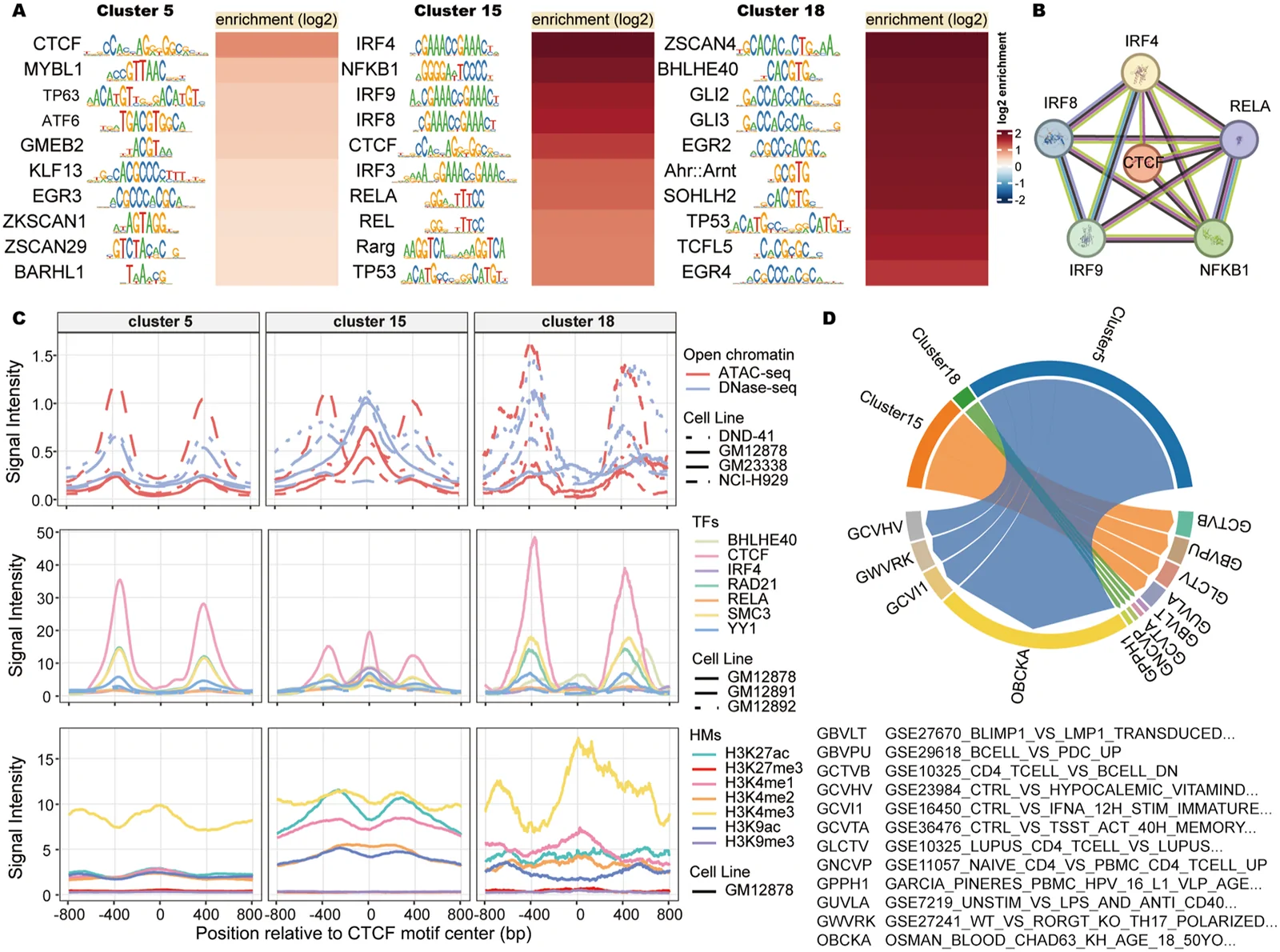
Functional characterization of Immune cell-enriched CTCF regulatory clusters. (A) De novo motif enrichment for key transcription factors in each cluster. Heatmaps show log2 enrichment scores. (B) PPI network for candidate co-factors associated with CTCF and IRF family TFs. (C) Epigenomic profiles centered on CBSs. The x-axis displays the distance from the motif center of CBSs, while the y-axis represents the signal intensity of chromatin accessibility or epigenetic modifications. The top shows chromatin accessibility signals across the profiled immune cell lines; the middle displays ChIP-seq signals of TFs; and the bottom presents ChIP-seq signals of six HMs. Distinct line styles correspond to different cell lines, and different colors indicate distinct epigenetic signals. (D) Chord diagram showing the top enriched immunologic gene sets from MSigDB C7. Cluster 5 is associated with acute interferon response and Th17 polarization; Cluster 15 with B cell vs. pDC and CD4 vs. B cell differentiation signatures; and Cluster 18 with memory CD4 + T cell and germinal-center B cell programs.

Finally, to elucidate the immunological functions of the ICM, we performed gene set enrichment analysis against the MSigDB C7 immunologic signatures collection (Fig 5D). Genes associated with the structure-oriented Cluster 5 are enriched in pathways of acute interferon response and Th17 polarization. Cluster 15 is strongly enriched for gene signatures that distinguish between different lymphocyte populations, such as B cells from pDCs, and CD4 + T cells from B cells. This result suggests an association between Cluster 15 and lymphocyte crosstalk and activation. The BHLHE40-associated Cluster 18 is specifically enriched in programs related to memory CD4 + T cells and germinal-center B cells, suggesting an association with adaptive immunity (S4 Table).

Taken together, the ICM delineates three distinct functional modes of CBSs in the immune cell lines included in our dataset, each showing further specialization within different cell lineages. A canonical CTCF motif-enriched, architecture-associated mode linked to acute interferon response and Th17 polarization (Cluster 5); an IRF/NF-κB-associated mode characterized by a triple-peak CTCF/cohesin ChIP-seq signal profile and linked to lymphocyte identity and activation (Cluster 15); and a BHLHE40-associated mode linked to memory CD4 + T-cell and germinal-center B-cell programs (Cluster 18).

### TF cooperation is associated with CTCF lineage functions

However, we observed that classifying CBSs by cell type alone cannot fully explain their functional diversity. Instead, we hypothesized that differences in TF motif enrichment reveal functionally relevant TF cooperation. Identifying these co-factors allows us to further correlate the clusters with four distinct functional dimensions. To test this, we integrated data derived from motif enrichment, GO profiling, and ChIP-seq signal profiles. This analysis revealed a consistent pattern: a cluster’s co-binding TF motifs are strongly associated with specific local histone/ATAC signatures and characteristic CTCF/cohesin ChIP-seq signal profiles. In short, the specific combination of co-binding TFs, CTCF/cohesin ChIP-seq signal profiles, and the local epigenetic environment is associated with differences in the regulatory functions of CBSs across contexts (Fig 6).

**Fig 6:**
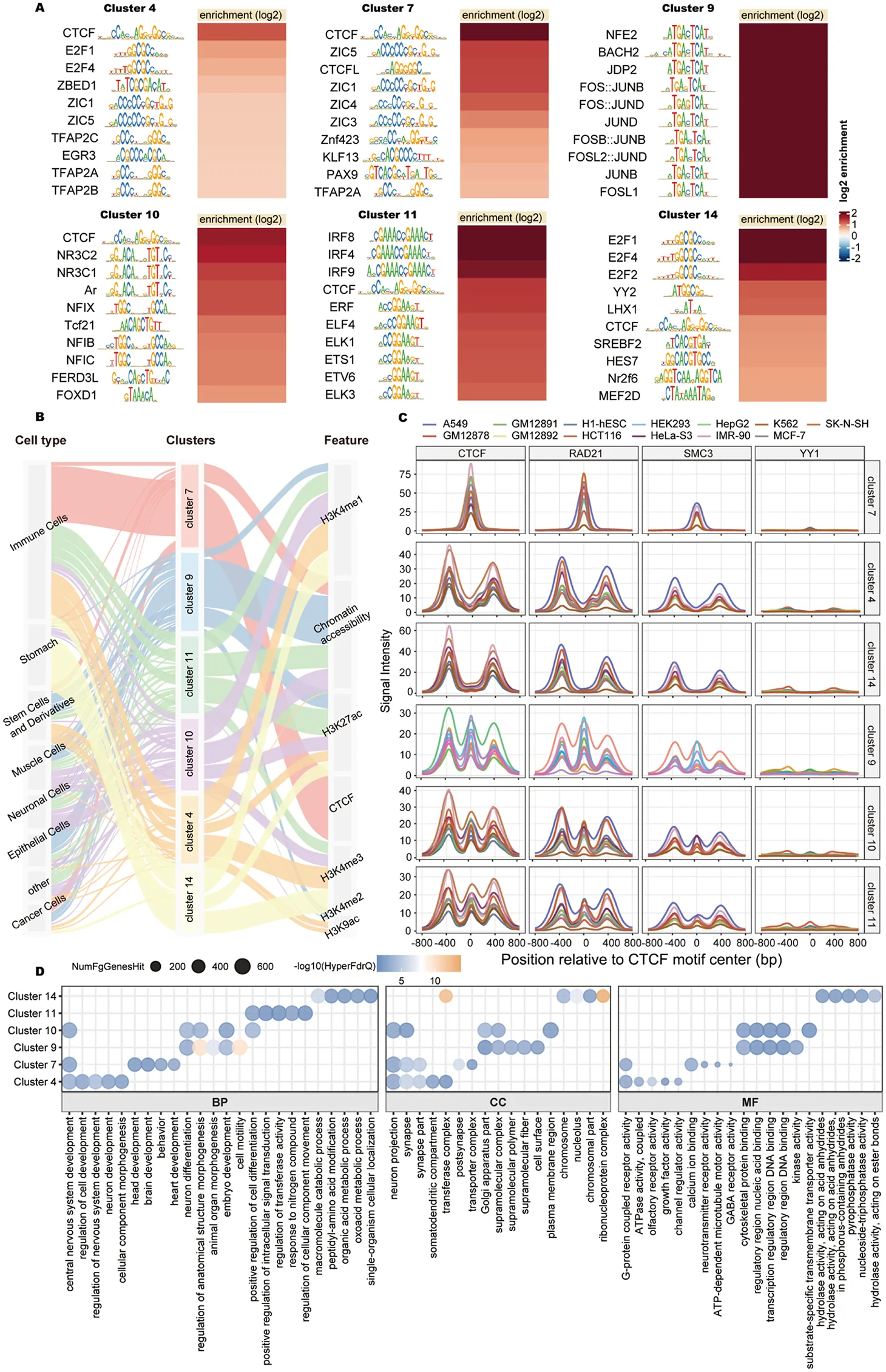
Co-binding factors associated with the functional diversity of CTCF cohorts. (A) De novo motif enrichment for key TFs in six clusters. Heatmaps show log2 enrichment scores. (B) Sankey diagram illustrating the dominant associations between cell types (left), CTCF clusters (center), and local epigenetic features (right), including H3K4me1/3, H3K27ac, and chromatin accessibility. (C) Profiles of CTCF, RAD21, SMC3, and YY1 ChIP-seq signals. The x-axis displays the distance from the motif center of CBSs, while the y-axis represents the ChIP-seq signal intensity for each transcription factor. Differently colored curves indicate distinct cell lines. (D) Gene Ontology (GO) analysis of biological processes for genes associated with each cluster.

Motifs identified through enrichment analysis implicated the corresponding TFs as potential partners in each cluster. We further evaluated this inference by integrating epigenetic signatures (Fig 6B), CTCF/cohesin ChIP-seq signal profiles (Fig 6C), and GO functional analysis (Fig 6D), revealing a series of highly consistent, lineage-associated regulatory modules. Although Clusters 4 and 14 (Divergent E2F Functions) are enriched for the cell-cycle regulator E2F, they are associated with distinct biological outcomes. Cluster 4, accessible in immune and epithelial lineages, is unexpectedly linked to central nervous system development, suggesting potential E2F re-deployment associated with neural gene expression in specific developmental contexts. Conversely, Cluster 14 is particularly prominent in stomach cancer cells and is associated with macromolecule metabolism. This may reflect E2F-driven metabolic reprogramming—a hallmark of cancer proliferation—and suggests that the same candidate TF partner may be associated with divergent roles depending on the cellular milieu. Cluster 7 (Structural Anchor with Neural Potential) is defined by ZIC/CTCFL motifs and strong GO enrichment for the central nervous system and brain development. However, the CTCF ChIP-seq signal within this cluster is predominantly from lymphoblastoid cells, where it displays a classic single-peak profile. Therefore, we could define Cluster 7 as a group of constitutive anchors whose CBSs are associated with regulatory potential in neural development. Cluster 9 (AP-1-driven Epithelial Remodeling): Dominated by AP-1 (JUN/FOS) motifs and epigenomic signals from epithelial cells, this cluster’s functions are linked to morphogenesis and cell motility. It probably represents AP-1-driven biological processes related to cell shape and movement, which are important for development and disease in epithelial tissues. Clusters 10 & 11 (Specialized Differentiation Hubs): Perhaps the most striking contrast is found between Clusters 10 and 11. While both are associated with differentiation, they exhibit fundamentally different regulatory features. Linking Nuclear Receptor (NR3C) motifs to neuronal and muscle lineages, Cluster 10 is associated with the physical architecture of differentiation. Detailed analysis (Fig 6D) reveals GO enrichment not just for neuron differentiation, but specifically for neuron projection, synapse formation, and Golgi-mediated transport. This suggests that Cluster 10 may support the structural machinery required to build a neuron. In contrast, Cluster 11, associated with Interferon Regulatory Factors (IRFs), may act as a dynamic signaling interface. Enriched for intracellular signal transduction and regulation of transferase activity, this cluster may represent a responsive node, associated with external stimuli (e.g., nitrogen response) and the kinase cascades involved in immune and stem cell fate decisions.

Collectively, the evidence indicates that: (1) Enriched TF motifs could identify candidate CTCF partners; (2) The local epigenetic modification environment provides the biochemical context (e.g., promoter vs. enhancer); and (3) The resulting CTCF/cohesin ChIP-seq signal profiles may be associated with distinct topological features. Thus, the specific combination of candidate co-binding factors, CTCF/cohesin ChIP-seq signal profiles, and chromatin states provides an integrative map for characterizing the regulatory diversity of CBSs (Fig 6A-6D).

### CTCF ChIP-seq signal profiles of CBSs associated with chromatin loops

In this study, we observed that the CBS clusters could be classified into three distinct CTCF ChIP-seq signal profiles (Fig 7A). The first profile exhibits a single-peak distribution (Clusters 1, 2, 7, 12 and 16), characterized by a typically narrow and sharp peak. The second profile exhibits a double-peak distribution (Clusters 4, 5, 14, and 18), with two adjacent maxima located at approximately ±400 bp. The third profile exhibits a triple-peak distribution (Clusters 3, 6, 8, 9, 10, 11, 13, 15, and 17), with a central peak flanked by two satellite peaks. Furthermore, we compared canonical CTCF motif-matching significance among the three peak-profile categories. Single-peak CBSs showed the strongest motif matches, followed by triple-peak CBSs, whereas double-peak CBSs showed the weakest motif matches (S5A Fig). These results indicate that CTCF ChIP-seq peak morphology is associated with canonical motif strength.

**Fig 7:**
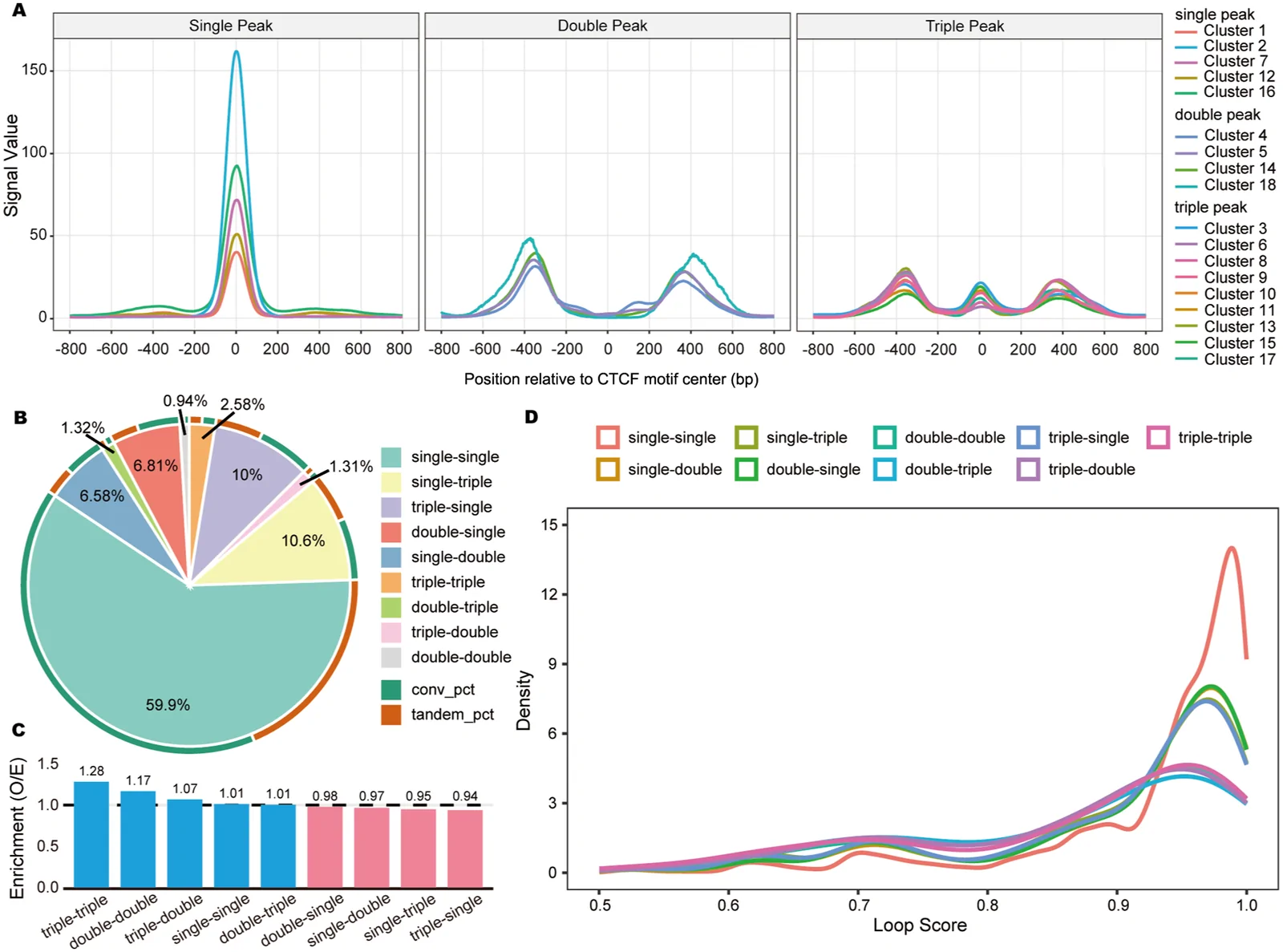
Peak patterns of CBSs correlate with chromatin loop pairing. (A) Average CTCF ChIP-seq signal profiles centered on CBSs, the x-axis displays the distance from the motif center of CBSs, while the y-axis represents the ChIP-seq signal intensity, categorized into three distinct profiles: Single-peak (left), Double-peak (middle), and Triple-peak (right). Representative clusters for each class are indicated in the legend. (B) Proportions of high-confidence chromatin loops classified by the combination of peak pattern at two anchors. (C) Observed-to-Expected (O/E) enrichment ratios for different combinations of anchor peak pattern. The dashed line indicates random expectation (O/E = 1). (D) Density distribution of loop interaction scores (Loop Score) for chromatin loops formed by different combinations (e.g., Single-Single, Single-Double).

To determine whether these profiles were reproducible rather than incidental features of the integrated dataset, we examined the CTCF ChIP-seq signal distributions of each cluster using independent cell-line datasets. The three profiles were also observed in K562, A549, and HepG2 cells (S6A Fig). We further selected representative CBSs from each category and examined their local CTCF binding signal distributions in these independent cell lines. These loci exhibited signal configurations consistent with their assigned peak categories, although the relative peak intensities varied among cell types (S6B Fig). Together, these results support the robustness of our profile-based classification. This classification prompted us to investigate whether the different signal profiles are associated with distinct roles in 3D genome organization.

Using a high-confidence set of chromatin loops from 13 human cell lines, we analyzed the link between peak pattern and loop organization. We classified each loop based on the peak shapes at both anchors, focusing on loops where both anchors contain a strong CTCF motif. The results revealed several key patterns. Firstly, the match of loop anchors was not random. For example, an anchor with a single peak preferentially pairs with another single-peak anchor, accounting for nearly 60% of all loops, followed by pairing with triple-peak anchors (10.6%) and double-peak anchors (6.58%) as shown in Fig 7B. However, to correct for the high abundance of single peaks, we calculated the Observed-to-Expected (O/E) enrichment ratios (Fig 7C). Remarkably, this revealed a strong preference for homotypic pairing among complex peaks. Triple-Triple pairs showed the highest enrichment (O/E = 1.28), followed by Double-Double pairs (O/E = 1.17), while heterotypic combinations (e.g., Single-Triple) were depleted (O/E < 1). This suggests that anchors with similar topological features are structurally more compatible for loop formation. Secondly, the peak forms at the two anchors are strongly correlated with loop strength. Loops anchored by two single-peak CBSs showed significantly higher interaction scores than other loop categories. More generally, loops with at least one single-peak anchor tended to be stronger than those formed exclusively by multi-peak CBSs (Fig 7D). Finally, directional analysis of motifs at the loop anchors revealed a distinct bias, with Anchor1 preferring the forward direction while Anchor2 favoring the reverse direction (S5B Fig), which confirms the abundance of the chromatin loops with canonical convergent CBS motif orientation. Therefore, in addition to motif orientation, the peak profiles of CBS clusters represent an additional feature associated with chromatin loop organization.

In summary, our analyses identified associations between aggregate CTCF ChIP-seq signal profiles and chromatin-loop properties. The single-peak profile was more frequently observed among CBSs anchoring stronger and higher-confidence chromatin loops. In contrast, double- and triple-peak CBSs were less frequently found at the highest-confidence loop anchors. These results suggest that CBSs exhibiting single-peak, high-occupancy CTCF binding may be preferentially associated with stronger loop anchors. Conversely, complex multi-peak cluster profiles were associated with less frequent anchoring of the highest-confidence loops. The underlying mechanisms remain to be determined, and the relative contributions of local chromatin context, cell-type-specific CTCF occupancy, and potential technical variation in data processing, alignment, and peak-summit assignment across datasets require further investigation.

## Discussion

In this study, we explored the extensive functional diversity of CBSs across diverse cell types. Leveraging epigenomic features predicted by the pretrained Sei model, we systematically classified over 230,000 CBSs into 20 clusters via graph-based clustering and subsequently organized these clusters into higher-order function-associated modules based on downstream annotations. Functionally, these clusters were associated with distinct chromatin architectural features, lineage-associated transcriptional programs, regulatory circuits, and CTCF ChIP-seq signal profiles. Our results support the dual roles of CTCF as a genome structural anchor and as a versatile regulator with functions that vary across cellular contexts [14,40,41]. S5 Table provides a hierarchical summary of the features, candidate co-factors, established knowledge, new insights, and supporting evidence for each DeCTCF cluster and module.

While previous studies have primarily focused on the conserved roles of CBSs at TAD boundaries and loop anchors [42], our findings indicate that a substantial fraction of the CTCF binding landscape is strongly cell-type-associated. This variation is associated with candidate partnerships involving lineage-associated TFs. For example, the candidate cooperation of the ZIC family with CTCF in stem cells, IRF/NF-κB in immune cells [23,43,44], and AP-1 in epithelial contexts illustrates how CTCF binding adapts to diverse cellular environments. These context-associated modules are often missed by motif-based analyses alone, highlighting the utility of our integrative computational framework, which leverages epigenomic features predicted by the pretrained Sei model to characterize complex sequence-associated regulatory patterns [35,45]. Compared with direct annotation based on predefined individual features, the Sei-derived representation provides a unified, high-dimensional description of sequence-predicted regulatory potential, enabling unsupervised identification of combinatorial patterns across CBSs. We subsequently used direct motif, epigenomic, cCRE, GO, and chromatin-loop annotations to interpret the resulting clusters, while recognizing that predicted features do not replace experimental measurements and may inherit biases from the model’s training data.

Our analysis also reveals a potential link between CTCF ChIP-seq signal profiles and chromatin architecture. We found that distinct CTCF ChIP-seq signal profiles—single-, double-, and triple-peak profiles, are associated with different chromatin-loop properties. Single-peak CBSs tend to mark stable, constitutive boundaries, whereas multi-peak CBSs appear to have greater architectural flexibility. This observation expands our understanding of current models of CTCF-cohesin loop extrusion by suggesting that CTCF ChIP-seq signal profiles provide an additional feature associated with 3D genome organization across cell types, although the molecular basis of the multi-peak profiles still requires further exploration.

Despite these advances, several limitations should be noted. First, our framework relies on bulk ChIP-seq datasets, which may mask regulatory heterogeneity at the single-cell level. Second, our clustering relies on predictive features from the Sei model rather than direct experimental measurements. While these computational inferences provide a scalable representation of regulatory potential, they cannot fully capture in vivo or cell-type-associated regulatory states. Furthermore, our downstream analyses may be influenced by biases in the model’s training data, potentially propagating them into the results. Finally, while our computational analyses nominate candidate modules and co-factors, functional validation using CRISPR-based perturbations or high-resolution chromatin conformation assays will be required to confirm their regulatory roles [46–49].

DeCTCF provides a scalable foundation for studying CTCF-mediated regulation in development and disease. Extending this framework to single-cell multi-omics data may facilitate dynamic tracking of CTCF-associated functions across differentiation trajectories [50,51], while integration with perturbation experiments will help establish causality. Moreover, as the general strategy underlying our approach is not specific to CTCF, it may be adapted to other architectural proteins and TFs [52], offering a strategy for decoding the modular organization of genome regulation [53]. In summary, we have developed a comprehensive framework that combines DNA sequence-based predicted epigenomic features with downstream analyses of candidate TF co-factors to characterize the functional diversity of CBSs. By providing a unified map of CTCF binding activities across cell types, we provide insights into genome architecture and lay the groundwork for systematically charting the sequence-based regulatory logic of the human genome.

## Materials and methods

### CTCF binding sequence representation and clustering

#### Sequence representation and feature extraction.

To construct a comprehensive catalogue of human CBSs, we first collected the CTCF ChIP-seq data in narrowPeak format of 118 distinct cell lines from the ENCODE and Gene Expression Omnibus (GEO) databases. To standardize the genomic reference, all peak positions were remapped to the GRCh38/hg38 assembly. For each peak, CTCF motifs were scanned using FIMO from the MEME Suite (https://meme-suite.org/meme/) using the CTCF position-weight matrix MA0139.1 from the JASPAR 2020 CORE vertebrate database (https://jaspar.elixir.no/). Both DNA strands were scanned using a uniform background model (A = C = G = T = 0.25), and motif occurrences with P < 1e-4 were retained. When multiple CTCF motif occurrences were detected within a peak, the occurrence with the highest FIMO score was selected. Its motif center was used as position 0, and a 600-bp genomic sequence (±300 bp) was extracted around this position. The resulting 600-bp CBSs from 118 cell lines were merged, and a custom deduplication process was applied: if two CBSs overlapped by more than 300 bp, only the CBS containing the higher-scoring FIMO motif occurrence was retained. This process yielded a high-confidence set of 236,552 unique CBSs.

This CBS set was then used as input for feature extraction. The pretrained Sei model was used as a fixed feature extractor in this study. Each 600-bp sequence was transformed into a 21,907-dimensional vector of predicted epigenomic features spanning transcription factor binding, histone modifications, and chromatin accessibility across diverse cellular contexts.

#### Graph-based clustering.

To partition the high-dimensional CBS embedding into biologically meaningful groups, we applied a graph-based clustering framework. First, principal component analysis (PCA) was applied to the 21,907-dimensional predicted feature vectors. To accommodate the size of the dataset, PCA was computed incrementally in batches of 10,000 CBSs. We retained the first 19 principal components, the smallest number of components reaching the prespecified cumulative explained-variance threshold of 95%.

Before graph construction, each retained component was standardized to zero mean and unit variance. A k-nearest neighbor (KNN) query was then performed in the standardized 19-dimensional PCA space using Euclidean distance and n_neighbors = 14. The value of n_neighbors was set to 14 to preserve local neighborhood structure while avoiding an overly dense graph. The neighbor relationships were converted into an undirected NetworkX graph, with the pairwise Euclidean distances stored as edge weights. Louvain community detection was implemented using the python-louvain best_partition function. The resolution was set to 1.0, the standard resolution setting used to avoid additional parameter tuning, and random_state was fixed at 42 for reproducibility. The resulting partition contained 20 clusters, which were fixed before downstream biological annotation.

### Functional annotation of CBS clusters

To interpret the biological significance of each cluster, we performed extensive functional annotations from sequence, genomic, cell type and epigenetic perspectives.

#### Motif enrichment analysis.

To identify key TFs associated with each CBS cluster, we performed motif enrichment analysis for each CBS cluster. Motif enrichment was calculated using the calcBinnedMotifEnrR function within the monaLisa R package (v1.10.1) [54]. The analysis utilized a comprehensive library of Position Weight Matrices (PWMs) for vertebrate TFs from the JASPAR 2020 database [55]. The enrichment of these motifs within the CBSs of each cluster (foreground) was statistically assessed against a background model composed of sequences sampled from the entire hg38 genome. For each cluster, we obtained log2 enrichment scores (log2enr) for all tested motifs. Motifs were then ranked in descending order based on their log2enr. The top 20 enriched motifs for each cluster were subsequently visualized, displaying the sequence logo for the corresponding TF alongside a corresponding heatmap bar representing enrichment scores.

#### Sequence conservation analysis.

To evaluate the evolutionary importance of CBSs within each cluster, we analyzed their inter-species sequence conservation. We utilized the PhastCons scores derived from a 100-way vertebrate genome alignment, accessed through the phastCons100way.UCSC.hg38 Bioconductor package. For each of the 20 clusters, the genomic coordinates were represented as GRanges objects. The gscores function from the GenomicScores R package was then used to compute an average PhastCons score for each CBS. Subsequently, we quantified the enrichment of highly conserved elements in each cluster. A CBS was defined as “highly conserved” if its average PhastCons score exceeded 0.8. We then calculated the proportion of these highly conserved CBSs in each cluster and used a hypergeometric test to assess the statistical significance of their enrichment relative to the proportion found in the total set of 236,552 CBSs (the background).

#### Genomic functions and regulatory elements annotation.

To characterize the genomic functions of each cluster, we performed genome annotation from two perspectives.

First, to learn the location distribution of CBSs across the genome, we annotated each cluster using the ChIPseeker R package (v1.40.1) [56,57] with GENCODE transcript annotations for the hg38 genome assembly. For each cluster, we calculated the percentage of CBSs overlapping with defined genomic elements, including promoters (±3 kb around transcription start site, TSS), 5’ UTRs, 3’ UTRs, exons, introns, and distal intergenic regions. The results were visualized as a stacked bar plot showing the location distribution of each cluster.

Second, to assess the functional potential of the CBSs, we evaluated the enrichment of candidate cis-Regulatory Elements (cCREs) from the ENCODE SCREEN database (hg38) [58] in each cluster. We focused on several key cCRE categories: promoter-like (PLS), proximal enhancer-like (pELS), distal enhancer-like (dELS), and others. For each cCRE category, we first quantified the number of CBSs that overlapped with the element’s coordinates by at least one base pair in a given cluster. To normalize for varying cluster sizes, the count of overlapping CBSs was divided by the total number of CBSs in that cluster, yielding an overlap proportion. To determine the statistical significance of the enrichment, a Z-score was calculated for each cluster *i*, using the formula:


$$\begin{array}{c} Z_{i}=\frac{P_{i}-\mu_{p}}{\sigma_{p}} \end{array}$$ (1)

Where *P_i_* represents the proportion of CBSs overlapping with a cCRE category in cluster *i*. *μ_p_* and *σ_p_* are the mean and standard deviation of the proportions for this category across all 20 clusters, respectively. The resulting Z-score represents the enrichment level of a specific regulatory element in each cluster.

#### Gene ontology (GO) analysis.

To investigate the biological functions associated with each cluster, we performed Gene Ontology (GO) enrichment analysis using the web-based Genomic Regions Enrichment of Annotations Tool (GREAT, version 4.0.4) [59]. For each cluster, the CBSs were supplied as query regions in BED format, with the human genome assembly (GRCh38) selected as the reference. To ensure a relevant comparison, the complete set of all 236,552 CBSs identified in this study was supplied as the custom background region. The association between genomic regions and target genes was determined using GREAT’s default model: “Basal plus extension”. In this model, a basal regulatory domain is first defined for each gene, ranging from 5.0 kb upstream to 1.0 kb downstream of its TSS. This domain is then extended up to a maximum of 1000 kb until reaching the basal domain of the nearest neighbouring gene.

To determine statistically significant associations, we first filtered GO terms by False Discovery Rate (FDR) q-value threshold of less than 0.05 (HyperFdrQ < 0.05). Subsequently, to identify the most relevant functions for each cluster, we ranked these significant terms in descending order based on the number of foreground genes associated with them (NumFgGenesHit). The top five terms from each of the three main GO categories (Biological Process, Molecular Function, and Cellular Component) were selected for downstream interpretation and visualization.

#### Immunologic signature enrichment.

For clusters showing a significant enrichment in immune cell types (Clusters 5, 15, and 18), we performed targeted gene enrichment analysis. The purpose was to specifically probe the immunologic functions of three clusters. As the limited number of CBSs in Cluster 18, this cluster is precluded a standard GO analysis.

First, to generate gene lists for each cluster, we associated the CBSs with their putative target genes using the ChIPseeker R package. A CBS was targeted to a gene if it was located within a ± 3000 bp window around the gene’s TSS, based on the TxDb.Hsapiens.UCSC.hg38.knownGene annotation. The background, or gene universe, for the analysis was determined based on the complete set of genes associated with all 236,552 CBSs.

Next, we performed an enrichment analysis against the MsigDB C7 immunologic signatures collection, retrieved using the msigdbr package. The significance of the overlap between our cluster-specific gene set and each C7 gene set was calculated using a hypergeometric test. We retained enrichment results that included at least two genes and had a p-value less than 0.05. The relationships between the three immune-related clusters and the top enriched immunologic gene sets were subsequently visualized as a Chord Diagram using the circlize R package.

#### Cell-type associations and enrichment analysis.

To systematically characterize the cell-type associations of the 20 clusters, we performed a multi-level analysis. First, to provide a quantitative measure of whether the CBSs in a cluster were broadly shared or restricted to a few cell lines, we introduced a cell-line specificity score (S). For any cluster, the score was defined as follows:


$$\begin{array}{c} S=1-\frac{1}{N}\sum_{i=1}^{N}\frac{k_{i}}{118} \end{array}$$ (2)

where *N* represents the total number of CBSs in the cluster, and the term *k*_*i*_ denotes the number of cell lines in which the *ith* CBS was observed. A score approaching 1 indicates high cell-line specificity, while a score approaching 0 indicates high conservation. The specificity scores for each cluster are presented in a bar chart in S2A Fig.

Next, to identify the specific cell lines and broader lineages associated with each cluster, we performed two-step enrichment Z-score analysis. The first step was conducted among 118 individual cell lines (results in S1 Fig). The second step was performed within 8 major cell lineages: Immune, Fibroblasts, Cancer, Epithelial, Neural, Muscle & Bone, Stem Cell, and Endothelial. A complete lineages list for the 118 cell lines is provided in S1 Table.

To measure the degree of enrichment at either the cell line or broader lineage level, we adopted the Z-score definition used for the cCRE analysis (formula 1). In brief, for each cell line or lineage, we calculated the proportion of the CBSs in each cluster. The Z-score for each cluster was obtained by normalizing its proportion using the mean and standard deviation of proportions across 20 clusters. This approach provided a quantitative index of the cell-type associations of each cluster, facilitating subsequent investigations of CBS functions in the cell lines included in our dataset.

#### Characterization of epigenetic landscape.

To characterize the local epigenetic landscape of each cluster, we performed two complementary analyses.

First, to visualize the average signal distributions, we curated 29 functional genomics datasets covering TF binding, histone modifications, and chromatin accessibility (ATAC-seq, DNase-seq) from the ENCODE database. For each data, the command-line utility bwtool [60] was used to extract signal intensities from the corresponding BigWig files. The signal range was defined as a 1600 bp window centered at the midpoint of the highest-scoring CTCF motif identified by FIMO (position 0) for each CBS in a given cluster. To provide occupancy-based evidence for the association between RFX5 and Cluster 3 CBSs, we also analyzed publicly available RFX5 ChIP-seq datasets from ENCODE for HeLa-S3, HepG2, IMR-90, and SK-N-SH cell lines and plotted the average RFX5 and cell-matched CTCF signals within ±2 kb of the FIMO-defined CTCF motif centers.

Second, to statistically identify the most prominent epigenomic features for each cluster, we leveraged the output of the Sei model. Using the model’s output files, which include feature contribution scores, we ranked all input epigenomic features based on their influence on the model’s predictions for each cluster’s sequences. The top 50 epigenomic features were considered the most informative features for characterizing each cluster. A comprehensive list of these top-contributing features for all clusters is provided in S3 Table.

#### Protein-protein interaction (PPI) network analysis.

To determine whether the TF motifs discovered in the monaLisa analysis correspond to proteins with experimentally verified interactions with CTCF, we performed a protein-protein interaction (PPI) analysis.

Using the STRING database (v12.0) [61], we constructed PPI networks with a query list composed of CTCF and other enriched co-binding TFs in our annotated clusters. To specifically focus on high-quality, validated connections, the search was designed to construct the network exclusively using the experimental evidence channel, with a minimum interaction score of 0.4 (indicative of medium confidence). The resulting experimental interaction network was subsequently inspected to identify reported experimental interactions between CTCF and the candidate co-factors.

### Analysis of 3D chromatin architecture

#### Chromatin TAD enrichment analysis.

To determine whether certain CBS clusters are preferentially located at chromatin domain boundaries, we performed an enrichment analysis using a set of conserved topologically associating domain (TAD) boundaries. The boundary dataset, comprising 14,289 stable TAD boundaries (defined as 100 kb windows) in the hg38 genome, was obtained from the research of McArthur et al [62]. We calculated the overlap between the CBSs in each cluster and TAD boundaries in this dataset. The enrichment of each cluster at these boundaries was then quantified using a Z-score. This calculation was analogous to the methodology used in the cCRE analysis (Formula 1)

#### Chromatin loop anchor analysis.

Based on their aggregate CTCF ChIP-seq signal profiles, we classified the 20 clusters into three descriptive categories: (1) Single-peak profile: presenting a single peak centered on the FIMO-detected CTCF motif (e.g., Clusters 1, 2, 7, 12 and 16); (2) Double-peak profile: two signal maxima approximately ±400 bp from the motif center, flanking a central dip (e.g., Clusters 4, 5, 14, 18); (3) Triple-peak profile: a central maximum flanked by two additional maxima (e.g., Clusters 3, 6, 8, 9, 10, 11, 13, 15, 17). We hypothesized that these three descriptive aggregate profiles were associated with different chromatin-loop properties.

To test this, we investigated the association between CTCF ChIP-seq signal profiles and chromatin looping. A comprehensive set of chromatin loops was first compiled by integrating data from 13 human cell lines available on the 3D Genome Browser. A custom Python script was then executed to create a detailed map linking each loop to signal-profile categories and motif orientations at its anchors. The script first used bedtools intersect to map CBSs assigned to the single-, double-, and triple-peak profile categories to the loop anchors. In cases where an anchor overlapped with multiple peaks, the CBS with the largest genomic overlap was assigned. In parallel, the DNA sequence underlying each peak was scanned for the canonical CTCF motif using FIMO, and each peak was annotated with the orientation and p-value of its strongest motif instance. Finally, each loop was annotated with a signal-profile category (single, double, triple, or none) and motif orientation for each anchor. Based on this annotation, each loop was categorized in two ways: 1) based on the signal-profile categories of its two anchors (e.g., “single-single,” “single-double,” etc.), and 2) for intra-chromosomal loops with CTCF motifs, by the relative orientation of CTCF motifs on both anchors (convergent, divergent, or tandem). The frequencies of these categories were subsequently used to elucidate the association between CTCF ChIP-seq signal profiles and chromatin-loop properties.

## Supporting information

S1 Table The detail information of 118 cell lines. (XLSX)

S2 Table The number of CTCF binding sequences (CBSs) in each module with specific functions. (XLSX)

S3 Table Rank of Mean importance of epigenetic features across 18 clusters. (XLSX)

S4 Table The detail information of MsigDB C7 annotation. (XLSX)

S5 Table Summary of major findings and supporting evidence across DeCTCF clusters. (XLSX)

S1 Fig Heatmap of cell-type enrichment z-scores for all CTCF clusters. The heatmap displays the enrichment of each of the 20 CTCF clusters (columns) across 118 individual human cell lines (rows). Cell lines are grouped into eight major lineages: Cancer, Endothelial, Epithelial, Fibroblasts, Immune, Neural, Muscle & Bone, and Stem Cell. The color of each cell represents the z-score, indicating the degree of enrichment (red) or depletion (blue) of a given cluster’s binding sites within a specific cell line, relative to the average distribution across all cell lines. This visualization highlights the cell-type and lineage-specific activity of distinct CTCF clusters.
(TIF)

S2 Fig Functional, evolutionary, and genomic characterization of 20 CTCF clusters. (A) Bar chart displaying the cell-line specificity score for each CTCF cluster. A score close to 1 indicates that the binding sites in a cluster are specific to a small number of cell lines, while a score close to 0, as seen for Cluster 2, indicates the sites are constitutively bound across most cell lines. (B) Bar chart showing the statistical significance of the enrichment for highly conserved sequences within each cluster. A sequence is defined as highly conserved if its PhastCons score is greater than 0.8. (C) Stacked bar plot illustrating the proportional distribution of CBSs across different genomic features for each cluster, as annotated by ChIPseeker. Features include promoters, UTRs, exons, introns, and distal intergenic regions. (D) Heatmap displaying the z-score enrichment of each cluster within different categories of candidate cis-Regulatory Elements (cCREs) from the ENCODE SCREEN database. Red indicates enrichment, and blue indicates depletion for element types such as promoter-like (PLS), proximal enhancer-like (pELS), and distal enhancer-like (dELS) elements.
(TIF)

S3 Fig UMAP embedding of four functional modules. (A) UMAP visualization highlighting the Chromatin Architecture Module (CAM), comprising Clusters 2, 3, and 16. (B) UMAP visualization highlighting the Stem Cell-enriched Module (SCM), comprising Clusters 1, 6, 8, 12, 13, and 17. (C) UMAP visualization highlighting the Immune Cell-enriched Module (ICM), comprising Clusters 5, 15, and 18. (D) UMAP visualization highlighting the TF Cooperations Module (TFCoM), comprising Clusters 4, 7, 9, 10, 11, and 14. In all plots, colored dots represent CTCF binding sites (CBSs) belonging to the specific module, while grey dots represent the background distribution of all other clusters.
(TIF)

S4 Fig CTCF and RFX5 ChIP-seq signal profiles at Cluster 3 CBSs across four human cell lines. Average CTCF (gray) and RFX5 (purple) ChIP-seq signals were calculated around Cluster 3 CBSs in HeLa-S3, HepG2, IMR-90, and SK-N-SH cells. Profiles are centered on the FIMO-defined CTCF motif centers and displayed within ±2 kb. Dashed vertical lines indicate the motif centers. CTCF and RFX5 signals are shown on independent y-axis scales to preserve their respective signal distributions. RFX5 enrichment around Cluster 3 CBSs across multiple cell lines supports an association between RFX5 occupancy and this CBS cluster.
(TIF)

S5 Fig CTCF motif-match significance, peak morphology, loop-anchor pairing, and motif orientation. (A) Distribution of canonical CTCF motif-match significance across single-, double-, and triple-peak CTCF binding sequences (CBSs). Motif-match significance was quantified as −log_10_(FIMO p-value) for the highest-scoring canonical CTCF motif identified at each CBS. Center lines indicate medians, boxes represent the interquartile range, and whiskers extend to 1.5 times the interquartile range. The numbers below the boxes indicate the number of CBSs in each category. (B) Association between CTCF peak morphology, loop-anchor pairing, and motif orientation. The central heatmap shows the percentage of chromatin loops assigned to each pairwise combination of peak-profile categories at Anchor 1 (rows) and Anchor 2 (columns). “None” indicates that the corresponding loop anchor did not overlap a CBS assigned to the single-, double-, or triple-peak profile category. The bar plots above and to the right show the percentages of anchors associated with CBSs carrying positive-strand (+) or negative-strand (−) CTCF motifs, as well as anchors without an assigned CBS, at Anchor 2 and Anchor 1, respectively. Anchor 1 shows a greater proportion of positive-strand motifs, whereas Anchor 2 shows a greater proportion of negative-strand motifs, consistent with the enrichment of convergently oriented CTCF motifs at chromatin-loop anchors.
(TIF)

S6 Fig Reproducibility assessment and representative genomic examples of the single-, double-, and triple-peak CTCF signal patterns. (A) CTCF signal profiles in K562, A549, and HepG2 cells, respectively, centered on the FIMO-defined CTCF motif center (±800 bp; gray dashed line). Each colored curve represents an individual cluster. Single-peak profiles include Clusters 1, 2, 7, 12, and 16; double-peak profiles include Clusters 4, 5, 14, and 18; and triple-peak profiles include Clusters 3, 6, 8, 9, 10, 11, 13, 15, and 17. (B) Genomic loci for each morphology class using GM12878 and an independent cell line. The examples correspond to Cluster 16 near *U2AF2* and Cluster 2 near *VARS1* for the single-peak class; Cluster 5 near *TRMT12* and Cluster 14 near *CDC37* for the double-peak class; and Cluster 3 near *ANXA2R-OT1* and Cluster 11 near *PARP14* for the triple-peak class. Magenta dashed lines indicate the FIMO-defined motif centers. CTCF tracks within each representative locus are displayed using the same y-axis scale. Representative loci overlapping the hg38 ENCODE blacklist were excluded. Profiles were generated without strand-oriented reversal.
(TIF)
