# Supplementary material for: DeCTCF: Decoding CTCF binding sequences by leveraging predicted epigenomic features
Source: PLoS Comput Biol. 2026 Oct 5;22(10):e1014848. doi: 10.1371/journal.pcbi.1014848 (PMC13652320; doi:10.1371/journal.pcbi.1014848)
Supplement: Supplementary file 1 [file pcbi.1014848.s013.docx]

**Manuscript ID:** PCOMPBIOL-D-26-00474

**Title:** DeCTCF: Decoding CTCF binding sequences by leveraging predicted epigenomic features

**Dear Dr. Libbrecht,**

We sincerely thank you for your careful evaluation of our manuscript and for the constructive comments. We have revised the manuscript and addressed all concerns raised. Below we provide a point-by-point response.

**Editor comment 1**

*The most important step of the proposed analysis concerns clustering of the CTCF binding sites (CBS), but no details are given beyond the use of "K-nearest neighbor (KNN) graph construction and Louvain community detection". Please describe this in sufficient detail so that a reader can replicate the analysis.*

**Response**

Thank you for this important comment. We agree that the clustering of CTCF binding sites (CBS) is a key step in our study, and we regret that the methodological details were insufficiently described in the original manuscript.

Briefly, CBS sequences are first encoded into high-dimensional representations using the Sei model. In the revised manuscript, we have added a detailed description of the feature construction (Materials and Methods, Section 1.1, Page 31, Lines 576–579) as follows:

These 21,907 dimensions correspond to distinct types of epigenomic profiles across diverse cell lines, meaning that this high-dimensional embedding effectively encapsulates the latent epigenetic features embraced in each sequence.

Subsequently, we reduce the dimensionality using principal component analysis (PCA), construct a k-nearest neighbor (KNN) graph, and apply the Louvain algorithm for graph-based clustering. In the revised manuscript, we have added a detailed description of the clustering procedure (Materials and Methods, Section 1.2, Page 32, Lines 581–593) as follows:

To partition the high-dimensional CBS embedding into biologically meaningful groups, we applied a graph-based clustering framework. First, to reduce dimensionality while preserving informative signals, principal component analysis (PCA) was performed on the feature vectors of 21,907-dimensional. To accommodate the scale of the dataset, we employed an incremental PCA approach, processing the data in batches of 10,000 samples. The top 50 principal components were retained, capturing over 95% of the total variance.

Prior to graph construction, the resulting components were standardized to unit variance. A k-nearest neighbor (KNN) graph was then constructed using k=14 and Euclidean distance as the similarity metric. Edge weights were defined based on pairwise distances, and an undirected graph was built accordingly. Using the Louvain algorithm, community detection was performed to identify clusters of sequences with similar regulatory profiles. The resolution parameter was set to 1.0, and a fixed random seed (random_state = 42) was used to ensure reproducibility. This procedure resulted in 20 clusters for downstream analysis.

**Editor comment 2**

*Discussion: Please explain the caveats due to relying on Sei predictions instead of direct experimental observations.*

**Response**

Thank you for this insightful suggestion. We agree that it is important to explicitly discuss the limitations associated with relying on predictive features rather than direct experimental observations.

Our clustering is based on features derived from the Sei model, which represent computational predictions of regulatory activity rather than direct measurements. As a result, these features may not fully capture in vivo, cell-type-specific regulatory states. In addition, we note that potential biases in the model’s training data may influence downstream analyses and could be propagated into our results. In the revised manuscript, we have added a paragraph in the Discussion to clarify these caveats (Page 30, Lines 542–547) as follows：

Second, our clustering relies on predictive features from the Sei model rather than direct experimental measurements. While these computational inferences provide a scalable representation of regulatory potential, they cannot fully present the reality in vivo cell-type-specific regulatory states. Furthermore, our downstream analyses may be influenced by biases in the model’s training data, potentially propagating them into the results.
