# Supplementary material for: DeCTCF: Decoding CTCF binding sequences by leveraging predicted epigenomic features
Source: PLoS Comput Biol. 2026 Oct 5;22(10):e1014848. doi: 10.1371/journal.pcbi.1014848 (PMC13652320; doi:10.1371/journal.pcbi.1014848)
Supplement: Supplementary file 2 [file pcbi.1014848.s014.docx]

**Response to Reviewers**

**Manuscript ID: PCOMPBIOL-D-26-00474R1**

**Title: DeCTCF: Decoding CTCF binding sequences by leveraging predicted epigenomic features**

Dear Dr. Ewing and Dr. Libbrecht,

Thank you for the opportunity to revise our manuscript, “DeCTCF: Decoding CTCF Binding Sequences by Leveraging Predicted Epigenomic Features” (Manuscript ID: PCOMPBIOL-D-26-00474R1). We sincerely appreciate your consideration of our work and the careful and constructive comments provided by the three reviewers.

We have carefully addressed all comments and substantially revised the manuscript and supplementary materials.

First, we have clarified that DeCTCF is not a newly developed deep learning model. Instead, it is an integrative computational framework that leverages epigenomic features predicted by the pretrained Sei model, together with downstream clustering and functional annotation analyses, to characterize the functional diversity of CTCF binding sequences.

Second, because several of our biological interpretations are based on computational analyses and data-derived associations rather than direct experimental validation, we have revised potentially causal or overly definitive statements where appropriate and more clearly acknowledged the corresponding limitations.

Third, in response to the reviewers’ requests for further investigation of the single-, double-, and triple-peak CTCF ChIP-seq signal profiles, we have added complementary computational analyses, including a comparison of canonical CTCF motif-match significance among the three profile categories, an assessment of the reproducibility of these aggregate signal profiles in additional cell lines, and representative genomic examples for each profile category.

We have also expanded the methodological details, improved data and code availability, and carefully revised the manuscript for clarity and accuracy.

Below, we provide a point-by-point response to all comments. The reviewers’ comments are reproduced in *italics*, followed by our responses and descriptions of the corresponding changes in the revised manuscript.

We believe that these revisions have substantially improved the rigor, clarity, and reproducibility of the study, and we hope that the revised manuscript is now suitable for publication in *PLOS Computational Biology*.

Sincerely,

Lu Chai, Yong Wang, and Lirong Zhang

on behalf of all authors

# **Response to Reviewer #1**

*The authors developed a framework DeCTCF based on deep learning models. Using this method, they analyze CTCF binding sequences from 118 human cell lines and reveal the context-specific mechanisms of CTCF sequences that underlie its regulatory diversity. However, there are still some minor issues that need to be addressed in order to enhance the quality of the manuscript.*

*1. In the manuscript, the authors repeatedly emphasizes “deep learning methods” or “deep learning framework,” which makes it seem like the authors developed a new deep learning model. However, this work mainly uses the embeddings generated by existing models such as Sei and builds an analysis framework based on them. I suggest revising the wording throughout the manuscript to make this point clearer and avoid overstating the methodological novelty.*

**Response:** We thank the reviewer for pointing this out. We agree that the wording used in the original manuscript was inaccurate and could have given the impression that DeCTCF is a newly developed deep learning model. We have revised the relevant content throughout the manuscript to clarify that DeCTCF is an integrative computational framework that leverages epigenomic features predicted by the pretrained Sei model. For example, “deep learning framework” was replaced with “integrative computational framework,” and “deep learning-based clustering” was replaced with “graph-based clustering of predicted epigenomic features.” Corresponding revisions were also made in the Abstract, Author Summary, Introduction, workflow description, Discussion, and Keywords. We appreciate the reviewer’s helpful suggestion.

*2. In Line 179, the author mentioned “… and establishes stable loops that overlap most TAD”. The word “establishes” is too arbitrary since most of these conclusions are based on computational associations rather than direct experimental validation. So, it is suggested that the author use more cautious words regarding the results of the theoretical analysis to make the statement more accurate.*

**Response:** We thank the reviewer for this helpful comment. We agree that the word “establishes” implies a causal relationship that cannot be supported solely by our computational analysis. We therefore revised the corresponding sentence in the Results subsection “CTCF shaping chromatin structure with distinct binding modes” to: “Here, CTCF binds directly to conserved loci through its canonical motif, is co-occupied by cohesin (RAD21/SMC3), and is associated with stable chromatin loop anchors enriched at TAD boundaries.” This revised wording describes an association rather than implying a causal mechanism. Similar overly definitive statements were also revised where appropriate throughout the manuscript. We appreciate the reviewer’s suggestion.

*3. The relationship between peak morphology and motif score could be further explored. Each CBS has a corresponding FIMO motif score. It would be helpful to compare motif scores among single-, double-, and triple-peak categories. This analysis may help explain whether different peak morphologies are related to motif strength.*

**Response:** We thank the reviewer for this helpful suggestion. Following the reviewer’s recommendation, we compared motif scores according matching significance among the single-, double-, and triple-peak categories. Motif-match significance was quantified as -log₁₀(FIMO p-value) for the highest-scoring canonical CTCF motif identified at each CBS. Single-peak CBSs showed the strongest motif matches, followed by triple-peak CBSs, whereas double-peak CBSs showed the weakest motif matches. These results indicate an association between CTCF ChIP-seq peak morphology and canonical motif strength. The new analysis appears in the Results subsection “CTCF ChIP-seq signal profiles of CBSs associated with chromatin loops” (first paragraph line 498-502) and in Fig. S5A and its legend.

*4. More details should be added in the Methods section. Some important details are missing or not clear in the Methods section. For example, the FIMO motif-scanning parameters, such as the motif database, version, and threshold, should be provided. The reason for choosing clustering parameters, such as the number of PCs, k in the KNN graph, and Louvain resolution, should also be explained more clearly. These details are important for reproducibility.*

**Response:** We thank the reviewer for emphasizing the importance of methodological reproducibility. We have expanded the Methods section to provide the requested parameters and rationale. For FIMO scanning, we now specify the use of the CTCF position-weight matrix MA0139.1 from the JASPAR 2020 CORE vertebrate database, a uniform background model, scanning of both DNA strands, and a significance threshold of p < 1 × 10⁻⁴. When multiple motif occurrences were identified within a peak, the occurrence with the highest FIMO score was retained. These details are provided in Materials and Methods, subsection 1.1, “Sequence representation and feature extraction” ((Lines 625–631)). For clustering, we clarify that 19 PCs were retained as the minimum number required to reach a cumulative explained variance of 95%; the KNN graph was constructed using Euclidean distance and k = 14 to preserve local neighborhood structure without creating an overly dense graph; and Louvain clustering was performed at the standard resolution of 1.0 with a fixed random seed of 42. These clustering details and their rationale are provided in subsection 1.2, “Graph-based clustering” (Lines 645–657).

*5. Some language and grammar issues should be carefully revised throughout the manuscript. For example, Line 64, “Human genome is estimated…” should be revised to “The human genome…”; Line 72, “Advent of high-throughput sequencing technologies…” should be revised to “The advent of…”; and Line 75, “topological boundaries” should be revised to “topologically associating domain (TAD) boundaries” for accuracy.*

**Response:** We thank the reviewer for pointing out these language and grammar issues. We have corrected all three examples as suggested. Specifically, the revised Introduction now reads “The human genome is estimated …” (Line 66), “The advent of high-throughput sequencing technologies …” (Lines 74–75), and “the boundaries of most topologically associating domains (TADs) …” (Lines 77–78). We have also carefully proofread the manuscript and corrected additional grammatical errors and unclear wording throughout. For example, we changed “partitions” to “partitioned” in the Fig. 1B legend (Line 139) and removed the duplicated “and” in Materials and Methods, subsection 3.2, “Chromatin loop anchor analysis.” The revised sentence now reads, “Finally, each loop was annotated with a signal-profile category (single, double, triple, or none) and motif orientation for each anchor” (Lines 833–834).

**Response to Reviewer #2**

*The manuscript by Chai et al. presents deCTCF, an integrative deep-learning framework designed to characterize the functional diversity of CTCF binding sites. The authors combined CTCF ChIP-seq datasets from 118 human cell lines, identifying 236,552 CTCF-binding sites, which were subsequently analyzed using deCTCF. Based on this analysis, the authors classified CTCF sites into 20 clusters organized into four major modules.
Given the multifunctional role of CTCF in vertebrate cells, efforts to classify CTCF binding sites based on their epigenetic signatures and functional properties are of interest. This is an active area of research, and several studies have already asked related questions.
Although the deCTCF generates a detailed classification of CTCF binding sites, I found it difficult to identify the major biological insights that emerge from the analysis. In addition, I have concerns regarding the interpretation of single-, double-, and triple-peak CTCF ChIP-seq patterns. The manuscript points biological significance to these observations, However, alternative technical explanations should be considered more carefully. In particular, these peak configurations may reflect technical artifacts arising from the integration and alignment of large numbers of ENCODE datasets generated across different laboratories and experimental conditions, including potential differences associated with genome-build conversions (hg19 to hg38) and data-processing pipelines.*

**General response:** We thank the reviewer for the careful and constructive evaluation of our manuscript. We agree that the principal biological insights needed to be presented more clearly and that the interpretation of the single-, double-, and triple-peak aggregate CTCF ChIP-seq signal profiles required greater caution. We have therefore clarified the major findings, added a summary table for the identified clusters and modules, included additional analyses and representative genomic examples to assess the reproducibility of the peak profiles, and more explicitly acknowledged possible technical explanations and the limitations of our computational analyses. Detailed responses are provided below.

*Major concerns to address:
1. Please provide a supplementary table listing all 236,552 CTCF-binding sites, together with their assigned clusters and modules. This resource would enhance the reproducibility of the study.*

**Response:** We thank the reviewer for this helpful suggestion. We have provided a complete dataset listing all 236,552 CTCF binding sites, together with their genomic coordinates and assigned clusters and modules. Because of its large size, the dataset has been deposited in Zenodo and is publicly available at https://doi.org/10.5281/zenodo.18057910. The corresponding DOI is also provided in the revised manuscript under “Availability of data” (Line 1119).

*2. The biological interpretation of the identified modules requires further clarification. Given the inclusion of 118 cell lines, it is unclear why only stem cell–specific and immune cell–specific modules were identified. In particular, the enrichment of six clusters within the stem cell module appears disproportionate considering that only three stem cell lines were included in the dataset of 118.*

**Response:** We thank the reviewer for raising this important point. We agree that the term “stem cell-specific module” could have incorrectly implied that these CBSs were exclusively present in stem cells. When constructing the unified catalogue of 236,552 CBSs, we generated a binary occupancy matrix recording the presence or absence of each CBS across the 118 cell lines. This matrix was not used for clustering. The 20 clusters were independently identified using epigenomic features predicted by Sei, after which the occupancy matrix was used to evaluate cell-line enrichment within each cluster.

Thus, the six clusters in the stem cell-enriched module do not correspond to six stem cell lines. Rather, they are independently identified CBS groups that subsequently showed prominent enrichment in the three profiled stem cell datasets while exhibiting distinct sequence, epigenomic, and functional characteristics. A given cell type may be enriched in multiple clusters, and a cluster may contain CBSs detected across multiple cell lines. To avoid misunderstanding, we have replaced “stem cell-specific” and “immune cell-specific” with “stem cell-enriched” and “immune cell-enriched,” respectively, and clarified that these terms indicate relative enrichment rather than exclusive presence in a particular cell type.

*3. The CTCF motif analysis presents an apparent inconsistency. While the Methods state that all 236,552 sites contain a canonical CTCF motif (lines: 568-574), clusters 3 and 16 are reported to lack canonical CTCF motifs (lines: 183-184, 201-202). This discrepancy should be resolved.*

**Response:** We thank the reviewer for identifying this apparent inconsistency. We agree that the original wording inaccurately conflated the occurrence of a canonical CTCF motif at individual CBSs with its relative enrichment at the cluster level. During construction of the CBS catalogue, each ChIP-seq peak was scanned using FIMO, and the highest-scoring canonical CTCF motif occurrence passing the specified threshold was used to define the CBS center. Thus, all 236,552 CBSs contain a FIMO-detected canonical CTCF motif. In contrast, the cluster-level analysis evaluated the relative enrichment of a broad collection of TF motifs against background sequences, with only the top-ranked motifs displayed. Therefore, the relatively low ranking of the CTCF motif in Clusters 3 and 16 indicates limited relative enrichment rather than the absence of the motif. We revised the manuscript as follows:

**①. Pages 9–11, Results subsection “CTCF shaping chromatin structure with distinct binding modes,” Cluster 3 paragraph (Lines 188–189 and 209–212):**

**The following sentences in the original manuscript**

“Cluster 3 contains 21,935 CBSs characterized by strong RFX5 motif enrichment and the absence of the canonical CTCF motif (Fig. 2B).”

and

“In this mode, CTCF binding facilitates transcriptionally poised higher-order structures without relying on the canonical motif or strong cohesin occupancy, thereby integrating developmental signals with 3D genome folding (Fig. 2A).”

**were revised to**

“Cluster 3 contains 21,935 CBSs characterized by strong RFX5 motif enrichment and limited relative enrichment of the canonical CTCF motif (Fig. 2B).”

and

“In this mode, CTCF binding may be associated with transcriptionally poised higher-order structures despite comparatively weak enrichment of the canonical CTCF motif and low cohesin occupancy, potentially linking developmental signals to 3D genome folding (Fig. 2A).”

**②. Pages 11–12, Results subsection “CTCF shaping chromatin structure with distinct binding modes,” Cluster 16 paragraph (Lines 213–215 and 232–234):**

**The following sentences in the original manuscript**

“Cluster 16 comprises 6,529 CBSs devoid of the canonical CTCF motif yet exhibiting significant enrichment of Gmeb1 and other high-GC TF motifs (Fig. 2B).”

and

“This versatile, TF-guided strategy allows CTCF binding to link genome architecture with essential nuclear functions, even without the canonical motif (Fig. 2A).”

**were revised to**

“Cluster 16 comprises 6,529 CBSs exhibiting significant enrichment of Gmeb1 and other high-GC TF motifs but comparatively weak enrichment of the canonical CTCF motif (Fig. 2B).”

and

“This potential TF-associated mode may link CTCF binding and genome architecture with essential nuclear functions, despite the limited relative enrichment of the canonical CTCF motif (Fig. 2A).”

**③. Pages 33–34, Materials and Methods, subsection 1.1 “Sequence representation and feature extraction,” first paragraph (Lines 625–636):**

**The following sentence in the original manuscript**

“For each peak, the location of the CTCF motif was identified by FIMO and served as the anchor point for CTCF binding.”

**was expanded to**

“For each peak, CTCF motifs were scanned applying FIMO from the MEME Suite using the CTCF position-weight matrix MA0139.1 from the JASPAR 2020 CORE vertebrate database. Both DNA strands were scanned using a uniform background model (A = C = G = T = 0.25), and motif occurrences with P < 1e-4 were retained. When multiple CTCF motif occurrences were detected within a peak, the occurrence with the highest FIMO score was selected. Its motif center was used as position 0, and a 600-bp genomic sequence (±300 bp) was extracted around this position. […] This process yielded a high-confidence set of 236,552 unique CBSs.”

**④. Pages 35–36, Materials and Methods, subsection 2.1 “Motif enrichment analysis,” first paragraph (Lines 664–674):**

**The following sentence in the original manuscript**

“To identify key TFs associated with each CBS cluster, we performed motif enrichment analysis for each CBS.”

**was revised to**

“To identify key TFs associated with each CBS cluster, we performed motif enrichment analysis for each CBS cluster.”

The revised paragraph further clarifies that motifs were evaluated against background sequences sampled from the hg38 genome, ranked according to their log2 enrichment scores, and that only the top 20 enriched motifs for each cluster were displayed.

These revisions explicitly distinguish the FIMO-based identification of a canonical CTCF motif at each individual CBS from the cluster-level comparison of relative motif enrichment.

*4. The definition of the x-axis coordinate (“distance from the center of CTCF binding sequence”) should be clearly explained in all figures. Specifically, it should be stated whether profiles are centered on the CTCF motif (specific orientation?) or the ChIP-seq peak summit or else.*

**Response:** We thank the reviewer for pointing out this ambiguity. All aggregate signal profiles are centered on the midpoint of the highest-scoring canonical CTCF motif identified by FIMO within each CBS, which is defined as position 0. The profiles are therefore not centered on the ChIP-seq peak summit. Although both DNA strands were scanned during motif identification, the signal profiles were plotted in genomic coordinate orientation without strand-based reversal. This definition is specified in Materials and Methods, subsection 1.1, “Sequence representation and feature extraction” (motif center as position 0), and subsection 2.7, “Characterization of epigenetic landscape” (signal window centered on the FIMO-defined motif midpoint). We also revised the corresponding captions and x-axis labels for Figs. 2F, 3C, 4B, 5C, 6C, 7A, and S4-S6.

*5. The reported single-, double-, and triple-peak CTCF patterns require stronger validation. These patterns may reflect technical variability in peak summit assignment across the 118 integrated ChIP-seq datasets rather than distinct biological classes. Representative genome browser examples and the complete list of sites assigned to each cluster would help support the authors’ interpretation.*

**Response:** We thank the reviewer for raising this important concern. We agree that technical variability across ChIP-seq datasets should be considered when interpreting the single-, double-, and triple-peak signal profiles. We clarify that these aggregate profiles were centered on the highest-scoring FIMO-defined CTCF motif rather than on the ChIP-seq peak summit, thereby reducing their direct dependence on variability in summit assignment.

To assess their reproducibility, we generated cluster-level CTCF signal profiles separately in K562, A549, and HepG2 cells and observed reproducible single-, double-, and triple-peak aggregate profiles across these cell-line-specific datasets (new additions: Fig. S6A). We also added representative genomic loci for each profile category using GM12878 and an additional cell line (new additions: Fig. S6B). Loci overlapping the ENCODE hg38 blacklist were excluded, and signal tracks within each locus were displayed using the same y-axis scale. In addition, the complete list of 236,552 CBSs and their cluster assignments is publicly available in Zenodo at <https://doi.org/10.5281/zenodo.18057910>.

We have also revised the manuscript to describe these categories as descriptive aggregate CTCF ChIP-seq signal profiles rather than distinct biological classes and have more explicitly acknowledged possible technical explanations and the need for further validation.

*6. Relatedly, the discussion of triple-peak patterns (Results, lines 310–321; Figures 2F, 3C, 4B, 5C, and 6C) appears speculative. The authors should demonstrate that these patterns are biologically meaningful and not artifacts arising from differences in ChIP-seq processing, alignment, or summit calling across datasets generated by different laboratories.*

**Response:** We thank the reviewer for raising this important concern. We agree that the biological interpretation of the triple-peak profiles in the original manuscript, particularly their proposed connection with the cohesin handcuff model, was speculative.

To assess the reproducibility of these profiles, we first clarified that the signal profiles were centered on the midpoint of the highest-scoring FIMO-defined CTCF motif rather than on the ChIP-seq peak summit. This clarification is provided in Materials and Methods, subsection 2.7, “Characterization of epigenetic landscape” (Pages 40–41, Lines 779–782).

We further examined cell-line-specific CTCF profiles in K562, A549, and HepG2 cells and observed reproducible single-, double-, and triple-peak aggregate profiles. This new analysis is presented in Fig. S6A and its legend (Pages 51–52, Lines 1101–1106). We also added representative genomic examples for each profile category using GM12878 and an independent cell line. These examples and the corresponding analysis details are presented in Fig. S6B and its legend (Page 52, Lines 1106–1112).

These analyses make variability in summit assignment alone less likely to explain the observed profiles, but they do not fully exclude other technical effects or establish a specific biological mechanism. We therefore revised the Results to consistently describe these patterns as aggregate CTCF ChIP-seq signal profiles and to present the handcuff model only as one possible interpretation requiring validation in independently processed datasets and direct experiments (Results subsection “Stem cell-enriched CTCF regulatory patterns,” Page 17, Lines 327–341).

We also explicitly acknowledged that local chromatin context, cell-type-specific CTCF occupancy, and technical variation in data processing, alignment, and peak-summit assignment across datasets may contribute to the observed profiles (Results subsection “CTCF ChIP-seq signal profiles of CBSs associated with chromatin loops,” Page 29, Lines 535–545). Finally, the Discussion now emphasizes that the molecular basis of the multi-peak profiles remains unresolved and requires further investigation (Page 32, Lines 587–595).

*7. The proposal that RFX5 recruits CTCF at cluster 3 sites is currently based primarily on motif enrichment analysis. Additional supporting evidence, such as analysis of available RFX5 ChIP-seq datasets, would strengthen this conclusion.*

**Response:** We thank the reviewer for this helpful suggestion. We agree that motif enrichment alone is insufficient to support the conclusion that RFX5 recruits CTCF at Cluster 3 sites. We therefore analyzed publicly available, cell-matched RFX5 ChIP-seq datasets from HeLa-S3, HepG2, IMR-90, and SK-N-SH cells. RFX5 ChIP-seq signals were elevated around Cluster 3 CBSs in all four cell lines, providing occupancy-based evidence for an association between RFX5 and Cluster 3 sites (New additions: Fig. S4).

We have also revised the manuscript to interpret RFX5 as a candidate co-factor associated with Cluster 3 rather than as a factor that recruits CTCF. We now explicitly state that these observational ChIP-seq data do not establish recruitment or directionality and that direct experimental validation would be required to determine the underlying mechanism.

# **Response to Reviewer #3**

*The authors collected 118 CTCF ChIP-seq datasets from 118 cell lines and predicted the 21,907-dimensional regulatory potential from peak sequences using the Sei model. To reduce the computational burden of downstream analyses, they subsequently reduced the dimensionality from 21,907 to 50 via PCA. Based on the reduced embedding, the authors constructed a graph using KNN and grouped the peaks into 20 clusters via the Louvain algorithm. For each cluster, they annotated enriched TF motifs, inter-species sequence conservation, overlap with regulatory elements, gene ontology (GO), immunologic signature enrichment (a specific GO category for clusters enriched in immune cell types), cell-type specificity, epigenetic landscape, chromatin TADs, and chromatin loops. Based on these annotations, the authors further categorized the 20 clusters into four groups: 3D chromatin architecture, stem-cell specific, immune cell-specific, and TF cooperation. The stem cell-specific group was further divided into those enriched with ZIC-family motifs and those exhibiting triple-peak patterns.
The classification of CTCF peaks into three categories based on signal shape may be interesting: classical single peaks, canonical double peaks, and non-canonical triple peaks. The authors observed that each peak type tends to interact preferentially with itself. Notably, loops containing at least one single-peak anchor appear stronger than other loops. They suggest that peak morphology could assist CBS orientations for loop structure prediction. Another finding is that the authors provide an explanation for non-canonical triple peaks through the handcuff model of cohesin.*

**General response:** We thank the reviewer for the careful evaluation of our manuscript and for the clear summary of our analytical framework and principal observations. We appreciate the reviewer’s constructive comments regarding the presentation of the core findings, the rationale for using Sei-predicted features, the annotation of the identified clusters and modules, and the organization of the figures. We have carefully revised the manuscript to address these concerns. For clarity, we have separated the reviewer’s comments into individual points below.

*Major Comment 1：The primary limitation of the study is the lack of emphasis on core findings. It is difficult to distinguish well-established CTCF knowledge from novel insights. A hierarchical summary table would greatly* *improve clarity.*

**Response:** We thank the reviewer for this helpful suggestion. We agree that the core findings and their relationship to established CTCF knowledge were not sufficiently clear in the original manuscript. We have therefore added a hierarchical summary table organized by module and cluster (new additions: Table S5). The table summarizes the defining observations, relevant established knowledge, principal contribution or new insight, and supporting evidence for each cluster, thereby distinguishing findings that consolidate established CTCF biology from the principal insights emerging from our analysis. We have also revised the overview of the Results to emphasize the major findings and enhancing clarity (refer to Table S5).

*Major Comment 2：Another concern is the rationale for using the Sei model to perform supervised clustering before downstream routine analyses. The authors should discuss the advantages and differences compared to direct annotation of CTCF peaks.*

**Response:** We thank the reviewer for raising this important point. We would like to clarify that Sei was not used to perform supervised clustering. The pretrained Sei model was used as a fixed feature extractor to transform each CBS into a 21,907-dimensional vector of predicted epigenomic features, without training or fine-tuning the Sei model in this study. PCA, KNN graph construction, and Louvain community detection were subsequently applied in an unsupervised manner, and the resulting clusters were fixed before downstream biological annotation.

Compared with directly annotating CTCF peaks using individual features such as motif content, genomic location, or overlap with experimentally defined regulatory elements, the Sei-derived representation provides a unified, high-dimensional description of sequence-predicted regulatory potential across diverse chromatin contexts. This approach enables CBSs to be grouped without predefining annotation categories and may capture combinations of regulatory features that are not evident from any single annotation. Direct motif, epigenomic, cCRE, GO, and chromatin-loop annotations were then used to interpret and evaluate the independently derived clusters.

We have revised the Methods and Discussion to clarify this rationale and have also emphasized that Sei-predicted features do not replace direct experimental annotations and may be influenced by biases in the model’s training data.

*Major Comment 3：Additionally, how were features such as cell-type specificity and ZIC family enrichment selected to group the 20 clusters? Are these characteristics more important or statistically significant than others?*

**Response:** We thank the reviewer for raising this important question. We clarify that cell-line enrichment, ZIC-family motif enrichment, and the other downstream annotations were not used to generate the 20 clusters. The clusters were identified through unsupervised graph-based clustering regarding of Sei-predicted epigenomic features and were fixed before downstream biological annotation.

The higher-order modules were subsequently organized as descriptive biological groupings based on the most prominent and consistently supported characteristics of the clusters, including cell-line enrichment, TF motif enrichment, epigenomic profiles, functional annotations, and chromatin-architecture associations. Cell-line and motif enrichment were evaluated using z-scores and log2 enrichment scores, respectively. However, we did not perform a formal feature-importance comparison across these different annotation types and therefore do not claim that cell-line enrichment or ZIC-family motifs are generally more important than the other characteristics. These features were highlighted because they represented recurrent patterns supported by multiple complementary analyses. The defining observations and supporting evidence for each cluster and module are summarized in the new Table S5.

*Minors:
The order of figure panels is a bit random. They does not match the order in which they are referenced in the text.
The source code at https://github.com/22246013-LuChai/DeCTCF is not complete. It lacks 1_sei_prediction.py.
Line 82: No space after [23].
Line 113 and 116: I suggest remove these Figure references from the introduction. They are not special.
Line 147: partitions -> partitioned.
Line 765: and and*

**Response:** We thank the reviewer for the careful reading of our manuscript and for these helpful suggestions. We have carefully rechecked the figure-panel references and corrected missing or inconsistent citations so that each panel is referenced at the relevant point in the text. The overall panel layouts were retained because they group complementary analyses and preserve visual clarity. We have also added the missing 1_sei_prediction.py script to the GitHub repository and updated the corresponding usage instructions. In addition, we removed the indicated figure references from the Introduction, inserted the missing space after reference [23], corrected “partitions” to “partitioned” in the Fig. 1B legend (Line 139), and removed the duplicated “and” in Materials and Methods, subsection 3.2, “Chromatin loop anchor analysis” (Lines 833–834). We appreciate the reviewer’s attention to these details.
