# Supplementary material for: DeCTCF: Decoding CTCF binding sequences by leveraging predicted epigenomic features
Source: PLoS Comput Biol. 2026 Oct 5;22(10):e1014848. doi: 10.1371/journal.pcbi.1014848 (PMC13652320; doi:10.1371/journal.pcbi.1014848)
Supplement: Peer review history

# Editor report: Decision Letter 0

**DOI:** 10.1371/journal.pcbi.1014848.r001

**Authors:** Maxwell Libbrecht

17 Mar 2026

DeCTCF: Decoding CTCF binding sequences by leveraging predicted epigenomic features

PLOS Computational Biology

Dear Dr. Zhang,

Thank you for submitting your manuscript to PLOS Computational Biology. After careful consideration, we feel that it has merit but does not fully meet PLOS Computational Biology’s publication criteria as it currently stands. Therefore, we invite you to submit a revised version of the manuscript that addresses the points raised during the review process.

Please submit your revised manuscript by May 17 2026 11:59PM. If you will need more time than this to complete your revisions, please reply to this message or contact the journal office at ploscompbiol@plos.org. When you're ready to submit your revision, log on to https://www.editorialmanager.com/pcompbiol/ and select the 'Submissions Needing Revision' folder to locate your manuscript file.

Please include the following items when submitting your revised manuscript:

* A letter that responds to each point raised by the editor and reviewer(s). You should upload this letter as a separate file labeled 'Response to Reviewers'. This file does not need to include responses to formatting updates and technical items listed in the 'Journal Requirements' section below.

* A marked-up copy of your manuscript that highlights changes made to the original version. You should upload this as a separate file labeled 'Revised Manuscript with Track Changes'.

* An unmarked version of your revised paper without tracked changes. You should upload this as a separate file labeled 'Manuscript'.

If you would like to make changes to your financial disclosure, competing interests statement, or data availability statement, please make these updates within the submission form at the time of resubmission. Guidelines for resubmitting your figure files are available below the reviewer comments at the end of this letter

We look forward to receiving your revised manuscript.

Kind regards,

Maxwell Wing Libbrecht, Ph.D.

Section Editor

PLOS Computational Biology

Maxwell Libbrecht

Section Editor

PLOS Computational Biology

**Editor Comments :**

Thank you for your submission. Your work appears to be of possible interest to PLoS Computational Biology. However, before I send it for review, please address a few issues:

The most important step of the proposed analysis concerns clustering of the CTCF binding sites (CBS), but no details are given beyond the use of "K-nearest neighbor (KNN) graph construction and Louvain community detection". Please describe this in sufficient detail so that a reader can replicate the analysis.

Discussion: Please explain the caveats due to relying on Sei predictions instead of direct experimental observations.

**Journal Requirements:**

1) Please provide an Author Summary. This should appear in your manuscript between the Abstract (if applicable) and the Introduction, and should be 150-200 words long. The aim should be to make your findings accessible to a wide audience that includes both scientists and non-scientists. Sample summaries can be found on our website under Submission Guidelines:

https://journals.plos.org/ploscompbiol/s/submission-guidelines#loc-parts-of-a-submission

2) Please upload all main figures as separate Figure files in .tif or .eps format. For more information about how to convert and format your figure files please see our guidelines:

https://journals.plos.org/ploscompbiol/s/figures

3) Some material included in your submission may be copyrighted. According to PLOSu2019s copyright policy, authors who use figures or other material (e.g., graphics, clipart, maps) from another author or copyright holder must demonstrate or obtain permission to publish this material under the Creative Commons Attribution 4.0 International (CC BY 4.0) License used by PLOS journals. Please closely review the details of PLOSu2019s copyright requirements here: PLOS Licenses and Copyright. If you need to request permissions from a copyright holder, you may use PLOS’s Copyright Content Permission form.

Please respond directly to this email and provide any known details concerning your material’s license terms and permissions required for reuse, even if you have not yet obtained copyright permissions or are unsure of your material’s copyright compatibility. Once you have responded and addressed all other outstanding technical requirements, you may resubmit your manuscript within Editorial Manager.

Potential Copyright Issues:

i) Figure 1. Please confirm whether you drew the images / clip-art within the figure panels by hand. If you did not draw the images, please provide (a) a link to the source of the images or icons and their license / terms of use; or (b) written permission from the copyright holder to publish the images or icons under our CC BY 4.0 license. Alternatively, you may replace the images with open source alternatives. See these open source resources you may use to replace images / clip-art:

- https://commons.wikimedia.org

- https://openclipart.org/.

ii) The following Figure contains a logo or branding: 1. We are not permitted to publish this under our CC-BY 4.0 license, even with permission. We ask that you please remove or replace it.

4) Please amend your detailed Financial Disclosure statement. This is published with the article. It must therefore be completed in full sentences and contain the exact wording you wish to be published.

1) State what role the funders took in the study. If the funders had no role in your study, please state: "The funders had no role in study design, data collection and analysis, decision to publish, or preparation of the manuscript.".

Note: If the reviewer comments include a recommendation to cite specific previously published works, please review and evaluate these publications to determine whether they are relevant and should be cited. There is no requirement to cite these works unless the editor has indicated otherwise.

**Figure resubmission:**

After uploading your figures to PLOS’s NAAS tool - https://ngplosjournals.pagemajik.ai/artanalysis, NAAS will process the files provided and display the results in the "Uploaded Files" section of the page as the processing is complete. If the uploaded figures meet our requirements (or NAAS is able to fix the files to meet our requirements), the figure will be marked as "fixed" above. If NAAS is unable to fix the files, a red "failed" label will appear above. When NAAS has confirmed that the figure files meet our requirements, please download the file via the download option, and include these NAAS processed figure files when submitting your revised manuscript.

**Reproducibility:**

To enhance the reproducibility of your results, we recommend that authors of applicable studies deposit laboratory protocols in protocols.io, where a protocol can be assigned its own identifier (DOI) such that it can be cited independently in the future. Additionally, PLOS ONE offers an option to publish peer-reviewed clinical study protocols. Read more information on sharing protocols at https://plos.org/protocols?utm_medium=editorial-email&utm_source=authorletters&utm_campaign=protocols

# Author comment: Author response to Decision Letter 1

**DOI:** 10.1371/journal.pcbi.1014848.r002

27 Mar 2026

Attachment Submitted filename: Response to Reviewers.docx

# Peer review: Decision Letter 1

**DOI:** 10.1371/journal.pcbi.1014848.r003

**Authors:** Adam Ewing, Maxwell Libbrecht

14 Jul 2026

DeCTCF: Decoding CTCF binding sequences by leveraging predicted epigenomic features

PLOS Computational Biology

Dear Dr. Zhang,

Thank you for submitting your manuscript to PLOS Computational Biology. After careful consideration, we feel that it has merit but does not fully meet PLOS Computational Biology’s publication criteria as it currently stands. Therefore, we invite you to submit a revised version of the manuscript that addresses the points raised during the review process.

Please submit your revised manuscript by Sep 13 2026 11:59PM. If you will need more time than this to complete your revisions, please reply to this message or contact the journal office at ploscompbiol@plos.org. When you're ready to submit your revision, log on to https://www.editorialmanager.com/pcompbiol/ and select the 'Submissions Needing Revision' folder to locate your manuscript file.

Please include the following items when submitting your revised manuscript:

* A letter that responds to each point raised by the editor and reviewer(s). You should upload this letter as a separate file labeled 'Response to Reviewers'. This file does not need to include responses to formatting updates and technical items listed in the 'Journal Requirements' section below.

* A marked-up copy of your manuscript that highlights changes made to the original version. You should upload this as a separate file labeled 'Revised Manuscript with Track Changes'.

* An unmarked version of your revised paper without tracked changes. You should upload this as a separate file labeled 'Manuscript'.

Kind regards,

Adam Ewing

Academic Editor

PLOS Computational Biology

Maxwell Libbrecht

Section Editor

PLOS Computational Biology

**Additional Editor Comments (if provided):**

**Reviewers' comments:**

Reviewer’s Responses to Questions

**Comments to the Authors:**

**Please note here if the review is uploaded as an attachment.**

Reviewer #1: The authors developed a framework DeCTCF based on deep learning models. Using this method, they analyze CTCF binding sequences from 118 human cell lines and reveal the context-specific mechanisms of CTCF sequences that underlie its regulatory diversity. However, there are still some minor issues that need to be addressed in order to enhance the quality of the manuscript.

1. In the manuscript, the authors repeatedly emphasizes “deep learning methods” or “deep learning framework,” which makes it seem like the authors developed a new deep learning model. However, this work mainly uses the embeddings generated by existing models such as Sei and builds an analysis framework based on them. I suggest revising the wording throughout the manuscript to make this point clearer and avoid overstating the methodological novelty.

2. In Line 179, the author mentioned “… and establishes stable loops that overlap most TAD”. The word “establishes” is too arbitrary since most of these conclusions are based on computational associations rather than direct experimental validation. So, it is suggested that the author use more cautious words regarding the results of the theoretical analysis to make the statement more accurate.

3. The relationship between peak morphology and motif score could be further explored. Each CBS has a corresponding FIMO motif score. It would be helpful to compare motif scores among single-, double-, and triple-peak categories. This analysis may help explain whether different peak morphologies are related to motif strength.

4. More details should be added in the Methods section. Some important details are missing or not clear in the Methods section. For example, the FIMO motif-scanning parameters, such as the motif database, version, and threshold, should be provided. The reason for choosing clustering parameters, such as the number of PCs, k in the KNN graph, and Louvain resolution, should also be explained more clearly. These details are important for reproducibility.

5. Some language and grammar issues should be carefully revised throughout the manuscript. For example, Line 64, “Human genome is estimated…” should be revised to “The human genome…”; Line 72, “Advent of high-throughput sequencing technologies…” should be revised to “The advent of…”; and Line 75, “topological boundaries” should be revised to “topologically associating domain (TAD) boundaries” for accuracy.

Reviewer #2: The manuscript by Chai et al. presents deCTCF, an integrative deep-learning framework designed to characterize the functional diversity of CTCF binding sites. The authors combined CTCF ChIP-seq datasets from 118 human cell lines, identifying 236,552 CTCF-binding sites, which were subsequently analyzed using deCTCF. Based on this analysis, the authors classified CTCF sites into 20 clusters organized into four major modules.

Given the multifunctional role of CTCF in vertebrate cells, efforts to classify CTCF binding sites based on their epigenetic signatures and functional properties are of interest. This is an active area of research, and several studies have already asked related questions.

Although the deCTCF generates a detailed classification of CTCF binding sites, I found it difficult to identify the major biological insights that emerge from the analysis. In addition, I have concerns regarding the interpretation of single-, double-, and triple-peak CTCF ChIP-seq patterns. The manuscript points biological significance to these observations, However, alternative technical explanations should be considered more carefully. In particular, these peak configurations may reflect technical artifacts arising from the integration and alignment of large numbers of ENCODE datasets generated across different laboratories and experimental conditions, including potential differences associated with genome-build conversions (hg19 to hg38) and data-processing pipelines.

Major concerns to address:

1. Please provide a supplementary table listing all 236,552 CTCF-binding sites, together with their assigned clusters and modules. This resource would enhance the reproducibility of the study.

2. The biological interpretation of the identified modules requires further clarification. Given the inclusion of 118 cell lines, it is unclear why only stem cell–specific and immune cell–specific modules were identified. In particular, the enrichment of six clusters within the stem cell module appears disproportionate considering that only three stem cell lines were included in the dataset of 118.

3. The CTCF motif analysis presents an apparent inconsistency. While the Methods state that all 236,552 sites contain a canonical CTCF motif (lines: 568-574), clusters 3 and 16 are reported to lack canonical CTCF motifs (lines: 183-184, 201-202). This discrepancy should be resolved.

4. The definition of the x-axis coordinate (“distance from the center of CTCF binding sequence”) should be clearly explained in all figures. Specifically, it should be stated whether profiles are centered on the CTCF motif (specific orientation?) or the ChIP-seq peak summit or else.

5. The reported single-, double-, and triple-peak CTCF patterns require stronger validation. These patterns may reflect technical variability in peak summit assignment across the 118 integrated ChIP-seq datasets rather than distinct biological classes. Representative genome browser examples and the complete list of sites assigned to each cluster would help support the authors’ interpretation.

6. Relatedly, the discussion of triple-peak patterns (Results, lines 310–321; Figures 2F, 3C, 4B, 5C, and 6C) appears speculative. The authors should demonstrate that these patterns are biologically meaningful and not artifacts arising from differences in ChIP-seq processing, alignment, or summit calling across datasets generated by different laboratories.

7. The proposal that RFX5 recruits CTCF at cluster 3 sites is currently based primarily on motif enrichment analysis. Additional supporting evidence, such as analysis of available RFX5 ChIP-seq datasets, would strengthen this conclusion.

Reviewer #3: The authors collected 118 CTCF ChIP-seq datasets from 118 cell lines and predicted the 21,907-dimensional regulatory potential from peak sequences using the Sei model. To reduce the computational burden of downstream analyses, they subsequently reduced the dimensionality from 21,907 to 50 via PCA. Based on the reduced embedding, the authors constructed a graph using KNN and grouped the peaks into 20 clusters via the Louvain algorithm. For each cluster, they annotated enriched TF motifs, inter-species sequence conservation, overlap with regulatory elements, gene ontology (GO), immunologic signature enrichment (a specific GO category for clusters enriched in immune cell types), cell-type specificity, epigenetic landscape, chromatin TADs, and chromatin loops. Based on these annotations, the authors further categorized the 20 clusters into four groups: 3D chromatin architecture, stem-cell specific, immune cell-specific, and TF cooperation. The stem cell-specific group was further divided into those enriched with ZIC-family motifs and those exhibiting triple-peak patterns.

The classification of CTCF peaks into three categories based on signal shape may be interesting: classical single peaks, canonical double peaks, and non-canonical triple peaks. The authors observed that each peak type tends to interact preferentially with itself. Notably, loops containing at least one single-peak anchor appear stronger than other loops. They suggest that peak morphology could assist CBS orientations for loop structure prediction. Another finding is that the authors provide an explanation for non-canonical triple peaks through the handcuff model of cohesin.

The primary limitation of the study is the lack of emphasis on core findings. It is difficult to distinguish well-established CTCF knowledge from novel insights. A hierarchical summary table would greatly improve clarity. Another concern is the rationale for using the Sei model to perform supervised clustering before downstream routine analyses. The authors should discuss the advantages and differences compared to direct annotation of CTCF peaks. Additionally, how were features such as cell-type specificity and ZIC family enrichment selected to group the 20 clusters? Are these characteristics more important or statistically significant than others?

Minors:

The order of figure panels is a bit random. They does not match the order in which they are referenced in the text.

The source code at https://github.com/22246013-LuChai/DeCTCF is not complete. It lacks 1_sei_prediction.py.

Line 82: No space after [23].

Line 113 and 116: I suggest remove these Figure references from the introduction. They are not special.

Line 147: partitions -> partitioned.

Line 765: and and

**********

**Have the authors made all data and (if applicable) computational code underlying the findings in their manuscript fully available?**

The PLOS Data policyrequires authors to make all data and code underlying the findings described in their manuscript fully available without restriction, with rare exception (please refer to the Data Availability Statement in the manuscript PDF file). The data and code should be provided as part of the manuscript or its supporting information, or deposited to a public repository. For example, in addition to summary statistics, the data points behind means, medians and variance measures should be available. If there are restrictions on publicly sharing data or code —e.g. participant privacy or use of data from a third party—those must be specified.

Reviewer #1: Yes

Reviewer #2: **No:**  I could find the data deposited to Zenodo: 10.5281/zenodo.18057910

Reviewer #3: None

**********

PLOS authors have the option to publish the peer review history of their article (what does this mean?). If published, this will include your full peer review and any attached files.

If you choose “no”, your identity will remain anonymous but your review may still be made public.

**Do you want your identity to be public for this peer review?** For information about this choice, including consent withdrawal, please see our Privacy Policy.

Reviewer #1: No

Reviewer #2: No

Reviewer #3: No

[NOTE: If reviewer comments were submitted as an attachment file, they will be attached to this email and accessible via the submission site. Please log into your account, locate the manuscript record, and check for the action link "View Attachments". If this link does not appear, there are no attachment files.]

**Figure resubmission:**

**Reproducibility:**

?>

# Author comment: Author response to Decision Letter 2

**DOI:** 10.1371/journal.pcbi.1014848.r004

5 Sep 2026

Attachment Submitted filename: Response_to_Reviewers_auresp_2.docx

# Peer review: Decision Letter 2

**DOI:** 10.1371/journal.pcbi.1014848.r005

**Authors:** Adam Ewing, Maxwell Libbrecht

23 Sep 2026

Dear Prof. Zhang,

We are pleased to inform you that your manuscript 'DeCTCF: Decoding CTCF binding sequences by leveraging predicted epigenomic features' has been provisionally accepted for publication in PLOS Computational Biology.

Before your manuscript can be formally accepted you will need to complete some formatting changes, which you will receive in a follow up email. A member of our team will be in touch with a set of requests.

Please note that your manuscript will not be scheduled for publication until you have made the required changes, so a swift response is appreciated.

IMPORTANT: The editorial review process is now complete. PLOS will only permit corrections to spelling, formatting or significant scientific errors from this point onwards. Requests for major changes, or any which affect the scientific understanding of your work, will cause delays to the publication date of your manuscript.

Should you, your institution’s press office or the journal office choose to press release your paper, you will automatically be opted out of early publication. We ask that you notify us now if you or your institution is planning to press release the article. All press must be co-ordinated with PLOS.

Thank you again for supporting Open Access publishing; we are looking forward to publishing your work in PLOS Computational Biology.

Best regards,

Adam Ewing

Academic Editor

PLOS Computational Biology

Maxwell Libbrecht

Section Editor

PLOS Computational Biology

***********************************************************

Reviewer’s Responses to Questions

**Comments to the Authors:**

**Please note here if the review is uploaded as an attachment.**

Reviewer #1: Accept

Reviewer #2: The authors have adequately addressed my previous concerns and questions. I therefore recommend this manuscript for publication.

Reviewer #3: I thank the authors for their thorough and thoughtful response to my previous comments. They have added Table S5 to distinguish their insights from well-established knowledge about CTCF. The only remaining question is why Sei-based clustering outperforms direct clustering based on biological annotations. However, proving this would require substantial effort. The authors have satisfactorily addressed all my other concerns and made appropriate modifications to the manuscript. The quality and clarity of the paper have improved significantly as a result of these revisions. I am now satisfied with the manuscript and recommend it for acceptance.

**********

**Have the authors made all data and (if applicable) computational code underlying the findings in their manuscript fully available?**

The PLOS Data policyrequires authors to make all data and code underlying the findings described in their manuscript fully available without restriction, with rare exception (please refer to the Data Availability Statement in the manuscript PDF file). The data and code should be provided as part of the manuscript or its supporting information, or deposited to a public repository. For example, in addition to summary statistics, the data points behind means, medians and variance measures should be available. If there are restrictions on publicly sharing data or code —e.g. participant privacy or use of data from a third party—those must be specified.

Reviewer #1: None

Reviewer #2: Yes

Reviewer #3: Yes

**********

PLOS authors have the option to publish the peer review history of their article (what does this mean?). If published, this will include your full peer review and any attached files.

If you choose “no”, your identity will remain anonymous but your review may still be made public.

**Do you want your identity to be public for this peer review?** For information about this choice, including consent withdrawal, please see our Privacy Policy.

Reviewer #1: No

Reviewer #2: No

Reviewer #3: No

# Editor report: Acceptance letter

**DOI:** 10.1371/journal.pcbi.1014848.r006

**Authors:** Adam Ewing, Maxwell Libbrecht

PCOMPBIOL-D-26-00474R2

DeCTCF: Decoding CTCF binding sequences by leveraging predicted epigenomic features

Dear Dr Zhang,

I am pleased to inform you that your manuscript has been formally accepted for publication in PLOS Computational Biology. Your manuscript is now with our production department and you will be notified of the publication date in due course.

The corresponding author will soon be receiving a typeset proof for review, to ensure errors have not been introduced during production. Please review the PDF proof of your manuscript carefully, as this is the last chance to correct any errors. Please note that major changes, or those which affect the scientific understanding of the work, will likely cause delays to the publication date of your manuscript.

Soon after your final files are uploaded, unless you have opted out, the early version of your manuscript will be published online. The date of the early version will be your article’s publication date. The final article will be published to the same URL, and all versions of the paper will be accessible to readers.

For Research, Software, and Methods articles, you will receive an invoice from PLOS for your publication fee after your manuscript has reached the completed accept phase. If you receive an email requesting payment before acceptance or for any other service, this may be a phishing scheme. Learn how to identify phishing emails and protect your accounts at https://explore.plos.org/phishing.

Thank you again for supporting PLOS Computational Biology and open-access publishing. We are looking forward to publishing your work!

With kind regards,

Sharmila Kamatchi

PLOS Computational Biology | Carlyle House, Carlyle Road, Cambridge CB4 3DN | United Kingdom ploscompbiol@plos.org | Phone +44 (0) 1223-442824 | ploscompbiol.org | @PLOSCompBiol
